# RNA helicase DDX5 regulates the translation and genomic replication of foot-and-mouth disease virus

**DOI:** 10.1128/jvi.01731-25

**Published:** 2026-01-30

**Authors:** Jin'en Wu, Sahibzada Waheed Abdullah, Pinghua Li, Xuefei Wang, Mei Ren, Yuanyuan Huang, Xianglong Guo, Shiqi Sun, Huichen Guo

**Affiliations:** 1 State Key Laboratory of Animal Disease Control and Prevention, College of Veterinary Medicine, Lanzhou University, Lanzhou Veterinary Research Institute, Chinese Academy of Agricultural Sciences 111658 , Lanzhou, China; 2 College of Veterinary Medicine, Gansu Agricultural University 74661, Lanzhou, China; 3 Livestock and Dairy Development (Extension) Department, Government of Khyber Pakhtunkhwa 364971, Peshawar, Pakistan; 4 College of Veterinary Medicine, South China Agricultural University 12526, Guangzhou, China; 5 Department of Animal Science, Tibet Agricultural and Animal Husbandry College, Nyingchhi, China; 6 Gansu Province Research Center for Basic Disciplines of Pathogen Biology, Lanzhou, China; University of Kentucky College of Medicine, Lexington, Kentucky, USA

**Keywords:** internal ribosome entry site (IRES), DDX5, foot-and-mouth disease virus (FMDV), IRES-driven translation, replication

## Abstract

The internal ribosome entry site (IRES) is a cis-acting structural element found in many viral mRNAs, which mediates cap-independent translation by recruiting various RNA-binding proteins and IRES trans-acting factors (ITAFs). Foot-and-mouth disease virus (FMDV), a significant member of the *Picornaviridae* family, contains a functional IRES element that contributes to viral protein translation and RNA synthesis. Here, we uncover a previously unrecognized mechanism in which DEAD-box RNA helicase 5 (DDX5) functions as a novel ITAF, inhibiting FMDV translation and viral RNA synthesis through two distinct strategies. First, DDX5 binds to the D4 domain of the IRES, suppressing FMDV IRES-driven translation by blocking the assembly of 80S ribosome. Second, DDX5 interacts with the viral RNA-dependent RNA polymerase 3D^pol^ and 3′UTR of FMDV, disrupting viral RNA synthesis. Conversely, the inhibitory effect of DDX5 was counteracted by viral precursor protein 3ABCD-mediated proteolysis of 3C^pro^. Furthermore, the functional importance of DDX5 in FMDV pathogenicity was further validated *in vivo* experiments. These findings enhance our understanding of how viruses exploit or antagonize cellular factors to regulate IRES-driven translation and provide new insights into translational control during viral infection.

**IMPORTANCE:**

Picornaviruses have evolved various strategies to compete and dominate host protein synthesis machinery, often bypassing cap-dependent mRNA translation. Foot-and-mouth disease virus (FMDV), a highly contagious member of the *Picornaviridae* family, is a globally significant pathogen responsible for severe epidemics in cloven-hoofed animals, posing substantial economic and agricultural threats. In this study, we identified DEAD-box RNA helicase 5 (DDX5) as a novel IRES trans-acting factor that plays a critical role in the translational regulation of FMDV. Specifically, DDX5 was found to negatively modulate FMDV IRES-driven translation and suppress viral RNA replication during infection. Furthermore, we elucidated a novel viral counteraction mechanism in which DDX5 is cleaved by the viral precursor molecule 3ABCD through proteolytic activity. These findings provide new insights into the complex interplay between viral and host factors, advancing our understanding of translational control during picornavirus infection and offering potential avenues for the development of antiviral strategies.

## INTRODUCTION

Foot-and-mouth disease virus (FMDV), a cytoplasmic RNA virus of the *Picornaviridae* family, is known to cause one of the world’s most destructive and highly contagious animal diseases (1, 2). Its genome is composed of an 8.3 kb positive-strand RNA, including a long 5′-untranslated region (5′UTR), a large open reading frame (ORF), a short 3′UTR, and a poly-A tail. The 5′ terminus of viral RNA genome is covalently linked to VPg (a viral protein 3B), which maintains the stability of the viral genome and promotes the viral RNA synthesis (3, 4). The poly-A tail at the 3′ terminus can mimic the host mRNA to enhance the viral RNA translation and replication efficiency (5). The FMDV genome encodes a large viral polyprotein of approximately 250 kDa, which is subsequently processed by the leader protein (L^pro^), 2A, and 3C protease (3C^pro^) to eventually yield mature viral proteins, including four structural proteins (VP1, VP2, VP3, and VP4) and eight nonstructural proteins (L^pro^, 2A, 2B, 2C, 3A, 3B, 3C^pro^, and 3D^pol^), as well as several precursor molecules, such as 3ABCD, 3ABC, and 3BCD. These proteins work together to create an optimal environment for viral replication and propagation (6, 7).

Viruses, owing to the limited coding capacity of their genomes, have evolved various strategies to seize control of cellular translation factors and ribosomes, which not only subvert cellular gene expression strategies but also selectively prioritize viral protein translation (5, 8). Picornavirus RNA, lacking a 7-methylguanosine cap at its 5′ end, cannot be recognized by the host’s canonical cap-dependent translation machinery. Instead, it employs internal ribosome entry site (IRES)-mediated translation to facilitate viral protein synthesis (9). The 5′UTR of FMDV genome comprises five functional elements: S fragment (S-frag), poly(C) tract, pseudoknot structures (PKs), cis-acting replication element (Cre), and IRES. The IRES element functions as a ribosomal binding pad, facilitating ribosome recruitment for IRES-dependent translation (10, 11). The IRES of FMDV is one of the most potent IRESs known to date for undergoing protein translation. Its translation mechanism primarily depends on canonical eukaryotic initiation factors (e.g., eIF3, eIF4A, eIF4B, and eIF4G) in conjunction with auxiliary factors known as IRES trans-acting factors (ITAFs). Despite lacking a complete life system, viruses have evolved sophisticated mechanisms to suppress host protein expression. These mechanisms typically involve the recruitment of host factors to assist and prioritize viral protein translation and replication. For example, nucleolin (NCL) and ribosomal protein L13 (RPL13) function as ITAFs by directly binding to specific regions of the IRES to enhance the assembly of translation initiation complexes and 80S ribosomes for optimal translation of viral mRNA (12, 13). Additionally, polypyrimidine tract-binding protein directly interacts with FMDV IRES and acts as an RNA chaperone to stabilize the viral IRES structure (14).

Apart from utilizing host proteins to facilitate viral protein translation and replication, FMDV has also been discovered to modify host factors by viral enzymatic cleavage to enhance viral propagation. FMDV proteases L^pro^ and 3C^pro^ have the ability to cleave eukaryotic initiation factors 4G and 5B (eIF4G and eIF5B), leading to a serious blockade of cellular capped mRNA translation (15, 16). Consequently, the cleavage will provide adequate supplies of materials and energy for the FMDV IRES-mediated translation process. The nuclear protein Sam68 interacts with domains II to IV of the FMDV IRES, but it has been confirmed that the cleavage of Sam68 by 3C^pro^ facilitates viral replication (17). Meanwhile, it has also been reported that the cellular ITAFs, such as heterogeneous ribonucleoprotein K and L (hnRNP K and L) and G3BP1, can directly interact with IRES and inhibit FMDV translation and replication, but these ITAFs were cleaved by viral proteases during FMDV infection (6). These results indicate that ITAFs have different functions in FMDV replication. Therefore, a deeper understanding of the virus-host interactions involved in IRES-mediated translation is of great significance for the development of effective antiviral reagents.

DDX5 (p68), a multifunctional DEAD-box RNA helicase, plays important roles in all biological processes of RNA, including pre-mRNA processing, splicing, transport and decay, ribosome assembly, and translation (18, 19). Besides, it has also been reported that DDX5 is closely associated with the replication of various viruses. For example, DDX5 acts as a positive regulator of Japanese encephalitis virus (JEV) RNA synthesis by binding to viral 3′UTR to promote JEV replication, but not translation (20). Additionally, DDX5 promotes vesicular stomatitis virus (VSV) infection via regulating N6-methyladenosine levels on the DHX58 and NF-κB transcripts to enhance RNA decay of antiviral genes in a YTHDF2-dependent manner (21). Conversely, DDX5 can also exert inhibitory effects on the replication of hepatitis B virus and myxoma virus (18). These results indicate that DDX5 plays key roles in mediating antiviral infection or immune evasion in host cells. However, the current understanding of the role of DDX5 in the replication of picornavirus remains poorly understood.

In this study, we identified DDX5 as a critical cellular protein that inhibits FMDV translation and replication through its interaction with the FMDV IRES and 3D^pol^. Further investigation revealed that DDX5 blocks the assembly of the 80S ribosome, thus preventing the translation of IRES-driven FMDV mRNA. Additionally, we also found that the interaction between DDX5 and 3D^pol^ suppresses viral RNA synthesis via interfering with FMDV 3D^pol^ activity. Conversely, DDX5-mediated inhibition was antagonized by viral precursor molecules 3ABCD. Hence, our findings reveal a previously unrecognized role of DDX5 in translational control during FMDV infection, providing new insights into the intricate interplay between host factors and viral replication mechanisms.

## MATERIALS AND METHODS

### Cells and viruses

Baby hamster kidney cells (BHK-21, ATCC CCL-10) and porcine kidney cells (PK-15, ATCC CCL-33) were cultured in Dulbecco’s modified Eagle’s medium (Gibco) supplemented with 10% fetal bovine serum (Gibco), 100 U/mL penicillin, and 100 µg/mL streptomycin. Cells were maintained at 37°C with 5% CO_2_. The FMDV strain O/China/99 (GenBank accession no. AF506822.2) was obtained from the OIE/National Foot-and-Mouth Disease Reference Laboratory (Lanzhou, China) and propagated in BHK-21 cells. Virus titers were determined using the TCID_50_ assay.

### Antibodies and reagents

The monoclonal antibodies, including anti-DDX5, anti-β-actin, anti-Flag, and anti-HA, were purchased from Abcam (Cambridge, MA, USA). The protein synthesis inhibitor cycloheximide (CHX) and secondary antibodies conjugated with horseradish peroxidase (HRP), Alexa Fluor 488 (AF488), and AF561 were obtained from Sigma-Aldrich (St. Louis, MO, USA). Polyclonal pig antiserum against FMDV was generated and stored in our laboratory. Transfection reagents, including Lipofectamine LTX and RNAiMAX, were purchased from Invitrogen (CA, USA). TRIzol, an RNA extraction reagent, was also obtained from Invitrogen.

### Plasmid construction

Full-length and truncated DDX5 plasmids were synthesized by Tsingke (Beijing, China). Genes encoding FMDV structural and nonstructural proteins were constructed as previously described (1). Viral precursors 3ABCD, 3BCD, and 3CD were amplified from FMDV cDNA and sub-cloned into pCMV-N-Flag vector. Viral cDNA fragments corresponding to 5′UTR, the S-frag, the Cre, the IRES, and 3′UTR of FMDV genome were amplified and cloned into the pcDNA3.1 vector. Bicistronic reporter plasmids were constructed as previously described (13), with the sequences of FMDV, EMCV, and PV IRES cloned into the psiCHECK-2 vector (Promega, WI, USA). All constructs were confirmed by DNA sequencing.

### *In vitro* transcription and biotinylated RNA-pulldown assay

DNA templates for *in vitro* transcription were linearized by BamHI. The transcripts of FMDV RNA fragments were generated using RiboMAX large-scale RNA production systems-T7 (Promega), and then labeled with biotin using Pierce RNA 3′ End Desthiobiotinylation Kit (Thermo Scientific). The RNA fragments were then purified with TRIzol and stored at −80°C fridge. The biotinylated RNAs were incubated with PK-15 cell extracts, and RNA-protein complexes were isolated using the Pierce Magnetic RNA-Protein Pull-Down Kit (ThermoFisher) following the manufacturer’s protocols.

### RNA immunoprecipitation and reverse transcription PCR (RT-PCR)

Lysates from FMDV-infected PK-15 cells were collected at 5 h post-infection (hp.i.), and incubated with protein G-Sepharose 4 Fast Flow (GE Healthcare, MA, USA) on ice for 1 h, and then centrifuged at 4°C to remove non-specific complexes. An equal quantity of lysates was incubated and rotated overnight at 4°C with rabbit anti-DDX5, control rabbit IgG, or a buffer that contained no antibody as a negative control. The pre-washed protein G beads were then added and incubated for 2–4 h at 4°C. The RNA immunoprecipitation complexes were collected by centrifugation at 3,000 × *g* for 5 min at 4°C and washed three times with lysis buffer. RNA was extracted with TRIzol, and RT-PCR was conducted using the One Step RT-PCR Kit (Takara) to detect FMDV IRES, ORF, and 3′UTR fragments, as well as RPS16 and GAPDH. Primer sequences are listed in Table 1.

**TABLE 1:** Primers used in this study

No.	Primer name	Sequence (5′−3′)
1	FMDV-Fwd	CAAACCTGTGATGGCTTCGA
2	FMDV-Rev	CCGGTACTCGTCAGGTCCA
3	hGAPDH-Fwd	GTCCATGCCATCACTGCCACCCAG
4	hGAPDH-Rev	GCTGTTGAAGTCACAGGACACAAC
5	IRES-Fwd	CACAGGTTCCCACAACCGACAC
6	IRES-Rev	GCAGTGATAGTTAAGGAAAGGC
7	3D-Fwd	GTTGCTAGTGATTATGACTTGGAC
8	3′UTR-Rev	CTTACGGCGTCGCTCGCCTCAGAG
9	hRPS16-Fwd	TCGCAGCCATGCCGTCCAAGGGT
10	hRPS16-Rev	TCATTAAGATGGGCTCATCGGT
11	GAPDH-Fwd	TCCATGCCATCACGGCCACCCAG
12	GAPDH-Rev	ACTC TTGAAGTCGCAGGAGACAAC
13	sgRNA	CGATTTGGAGGAAGTAGGGC

### RNA interference and CRISPR-Cas9 technology

PK-15 cells were transfected with siRNAs (siNC or siDDX5) using RNAiMAX Reagent (Invitrogen) and incubated for 48 h. The siRNA sequences were synthesized by GenePharma (Shanghai, China) and are shown in Table 1. The gRNAs of DDX5 were designed on the Zhang laboratory website (http://crispor.gi.ucsc.edu/). The designed gRNAs were synthesized, annealed, and cloned into *BbsI* (NEB) sites in pX459 vector (Addgene #62988); then, PK-15 cells were transfected with pX459-gRNA plasmids using Lipofectamine LTX (Invitrogen). After 24 h transfection, puromycin (3 μg/mL) was added and selected for 5 days. The knockout efficacy was confirmed by Western blotting.

### Co-immunoprecipitation assay and Western blot

PK-15 cells were transfected with the indicated plasmids. After 24 h, cells were lysed with RIPA buffer on ice. Lysates were centrifuged at 15,000 × *g* for 20 min at 4°C, and an aliquot of supernatants was incubated with suitable antibodies. The immunoprecipitation complexes were added to pre-treated protein G beads and incubated at 4°C for 2–4 h. After the Sepharose beads were washed three times with lysis buffer containing a protease inhibitor, 50 μL of 1× SDS loading buffer was added into each sample. The proteins were transferred to a pure nitrocellulose blot membrane (PALL, Mexico), and blocked with 5% skimmed milk for 1 h. The membrane was incubated with appropriate primary and secondary antibodies. Signals were visualized using ECL reagents (Sigma-Aldrich).

### Polysome profile analysis

PK-15 or DDX5-knockout (DDX5-KO) cells, transfected with indicated plasmids for 24 h or infected with FMDV for 5 hp.i., were incubated with 0.1 mg/mL CHX for 15 min at 37°C to stabilize ribosomes. The cells were lysed using polysomal extraction buffer, and the lysates were centrifuged to remove cellular debris at 12,000 × *g* for 10 min at 4°C. The supernatants were transfected to 10%–50% sucrose gradient by centrifugation at 36,000 rpm at 4°C for 3 h in an SW40 Ti rotor (Beckman). Five hundred microliter fractions were collected from the uppermost layer, and the absorbance of the fractions was measured at 254 nm. The total RNAs in each fraction were extracted for reverse transcription-quantitative PCR (RT-qPCR) analysis. Each polysomal curve represents the mean absorbance values between replicates.

### Dual-luciferase reporter assay

PK-15 cells were seeded in 12-well plates until they reached approximately 70% confluence. Subsequently, the cells were transfected with either 1 μg of Flag-DDX5 or Flag-EV plasmid using Lipofectamine 2000. Eighteen hours post-transfection, a second round of transfection was conducted, introducing 1 μg indicated bicistronic constructs. Following an additional 24 h incubation period, cellular lysates were harvested using passive lysis buffer. The luciferase activities were then measured using the Dual-Luciferase Reporter Assay System (Promega) following the manufacturer’s specified protocols.

### Immunofluorescence assay

PK-15 cells were propagated on glass-bottomed cell culture dishes (NEST, China) and were infected with FMDV at specific times, or were transfected with indicated plasmids for 24 h. The cells were then fixed with 4% paraformaldehyde (PFA) for 20 min. After fixation, the cells were washed with PBST three times and permeabilized with 0.1% Triton X-100 for 20 min. The cells were blocked with PBS containing 1% bovine serum albumin (BSA) for 1 h at room temperature and were incubated with specific primary antibodies at 4°C overnight. Following three washes with PBST, the cells were stained with the corresponding AF488- or AF594-conjugated secondary antibodies for 1 h. After washing, cells were stained with DAPI (Molecular Probe, CA, USA) for 10 min at room temperature. Images were captured with a laser-scanning confocal microscope (Leica SP8, Solms, Germany).

### Fluorescence *in situ* RNA hybridization (FISH)

Complementary viral RNA sequence probes targeting FMDV strain O/China/99 (GenBank accession no. AF506822.2) were designed and synthesized with fluorophore conjugation by Servicebio Co. Ltd. (Wuhan, China). The RNA FISH of FMDV was performed according to the manufacturer’s instructions. Briefly, PK-15-WT and DDX5-KO cells were infected with FMDV at 1 MOI. At a designated time point post-infection, the cells were fixed with 4% PFA for 20 min, washed with PBS three times, and digested with proteinase K for 20 min and again washed with PBS three times (5 min for each). Next, target probes were pre-hybridized and hybridized in the pre-hybridization and hybridization buffers, respectively. Subsequently, the slides were washed with saline-sodium citrate at different concentrations. After washing, cells were stained with DAPI (Molecular Probe, CA, USA) for 10 min at room temperature. Finally, the slides were subjected to image acquisition with fluorescence microscope (OLYMPUS, IX73, Japan) and analyzed using Caseviewer 2.4.

### Viral challenge in suckling mice

Neonatal BALB/c mice, aged 2 days and organized into cohorts of 10 per experimental group, were procured from the Laboratory Animal Center of the Lanzhou Veterinary Research Institute, Chinese Academy of Agricultural Sciences (Lanzhou, China). The LD_50_ for the FMDV strain O/China/99 was determined using the Reed-Muench methodology. The neonatal mice were subsequently inoculated via subcutaneous administration in the cervical region with either 1.0 × 10^8^ PFU of the purified pAdM-shDDX5 or pAdM-shNC constructs, each diluted in 100 µL of PBS. Seventy-two hours after initial inoculation, a challenge was given to mice via the subcutaneous inoculation of a 20 LD50 dose of the FMDV strain O/China/99 or PBS. The survival rate for the neonatal mice was rigorously observed and documented for a 7 day period following the challenge protocol.

### Statistical analysis

All data are presented as mean ± standard error of the mean from at least two independent experiments. Statistical analyses were performed using Student’s *t*-test in GraphPad Prism 9 (GraphPad Software, La Jolla, CA, USA). Results were considered statistically significant at **P < 0.01.

## RESULTS

### DDX5 participates in FMDV propagation

**Fig 1:**
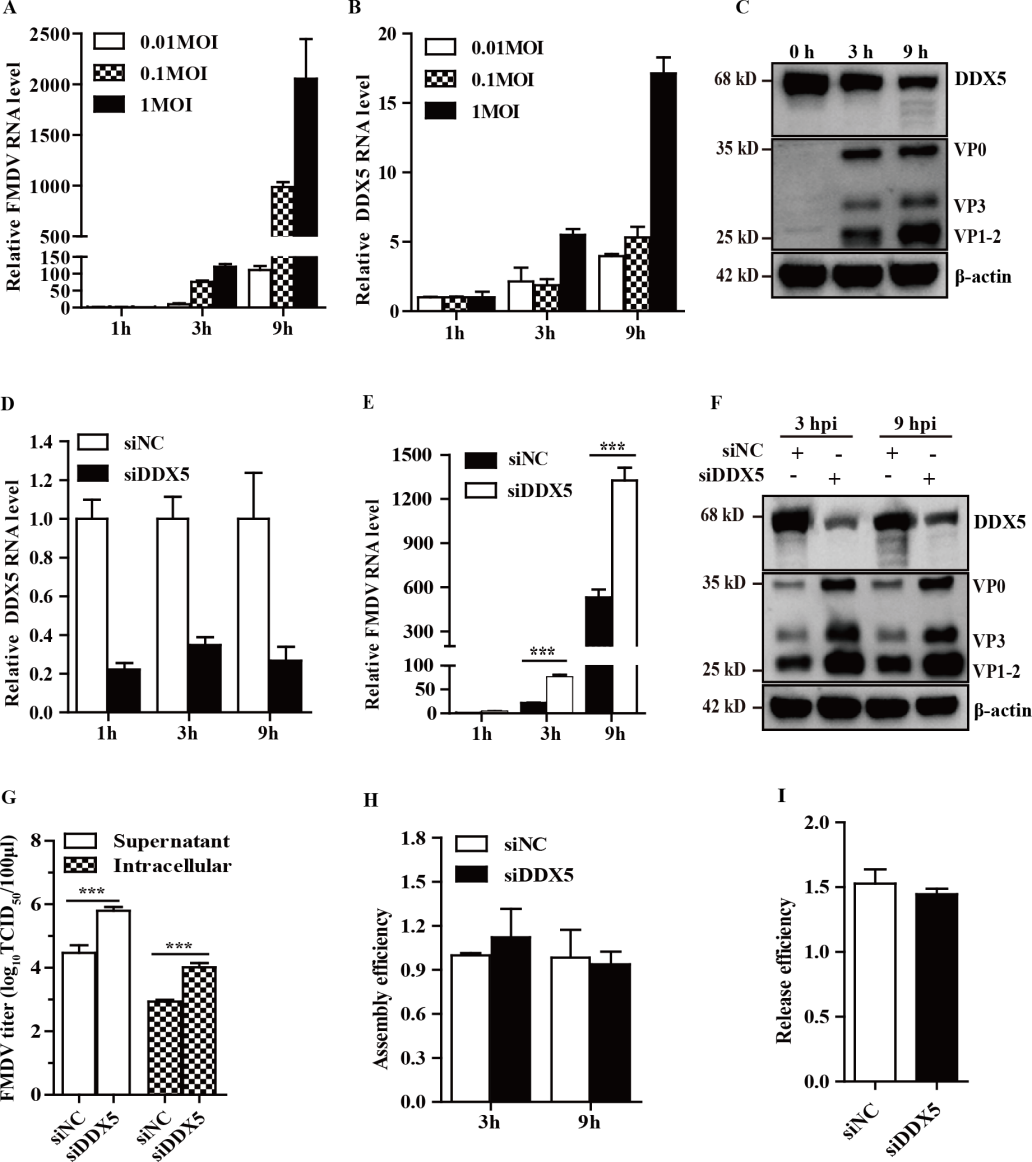
Participation of DDX5 in the FMDV life cycle. (**A–C**) PK-15 cells infected with FMDV at varying MOIs (0.01, 0.1, and 1 MOI) were harvested at the indicated time points. (**A and B**) The relative FMDV RNA levels (**A**) and DDX5 mRNA levels (**B**) were quantified by RT-qPCR. (**C**) The protein levels of FMDV (1 MOI) and endogenous DDX5 were detected by Western blotting at the indicated time points. (**D and E**) PK-15 cells were transfected with the indicated siRNAs and infected with FMDV. The intracellular DDX5 (**D**) and FMDV mRNA levels (**E**) were quantified by RT-qPCR at the indicated time points. (**F**) The DDX5 and FMDV structural protein expression levels were detected by Western blotting. (**G**) The FMDV titers in the supernatant and intracellular at 9 hp.i. were measured. (**H and I**) The efficiency of viral assembly and release was calculated. **P < 0.01 and ***P < 0.001.

To investigate the role of DDX5 in FMDV replication, we initially examined its expression dynamics following FMDV infection in PK-15 cells. As shown in Fig. 1A and B, FMDV infection led to a significant upregulation of DDX5 mRNA, but a concomitant reduction in DDX5 protein levels was observed (Fig. 1C). To explore whether DDX5 is required for FMDV replication, PK-15 cells were transfected with nonspecific siNC and specific siDDX5 and then were infected with FMDV. The siDDX5 efficiently downregulated DDX5 mRNA level (Fig. 1D) without obviously affecting cell viability. The knockdown of DDX5 led to a significant increase in the level of FMDV mRNA and structural proteins compared with that in siNC-transfected cells at 3 hp.i. and 9 hp.i. (Fig. 1E and F). Additionally, viral titers in intracellular and supernatant fractions were monitored. As shown in Fig. 1G, FMDV titer in siDDX5-transfected intracellular fraction was facilitated to a similar degree as the increase in supernatant compared with siNC-treated cells. To determine whether DDX5 knockdown affects virus assembly or release, the assembly efficiency (the ratio of FMDV RNA copy number in supernatant to intracellular FMDV RNA copies) and release efficiency (the ratio of FMDV titer in cell supernatant to intracellular FMDV titer) were calculated. As shown in Fig. 1H and I, the knockdown of DDX5 does not affect the assembly or release of FMDV.

### DDX5 negatively regulates FMDV proliferation

**Fig 2:**
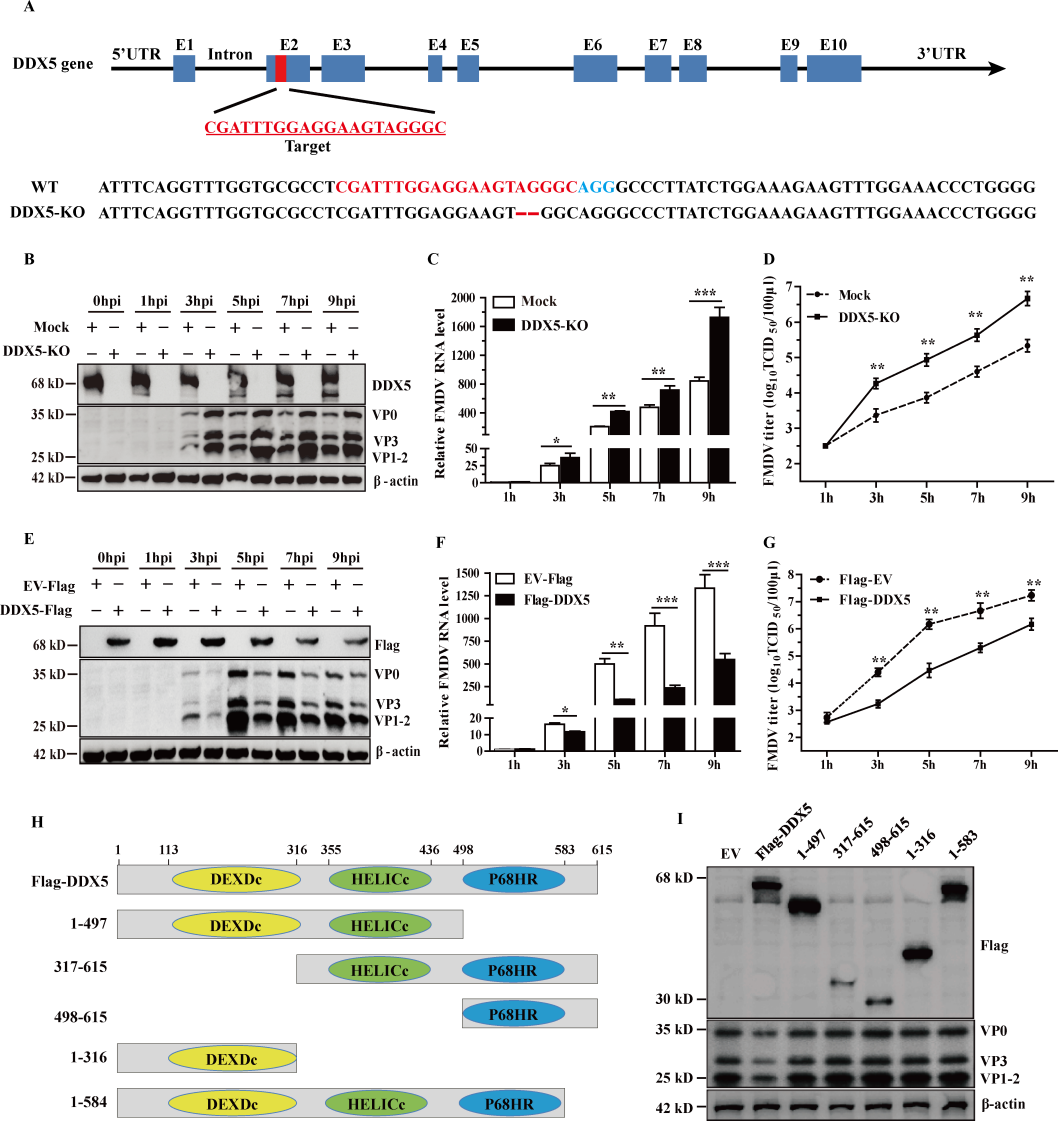
DDX5 negatively regulates FMDV proliferation. (**A**) Schematic illustrating CRISPR-Cas9 inactivation of porcine DDX5 locus. The guide RNA target sequence is shown in red, and the PAM is shown in blue. Two nucleotide deletions were found in DDX5-KO cells by sequencing. (**B**) The WT PK-15 and DDX5-KO cells were infected with FMDV at an MOI of 1, and samples were harvested at indicated time points. The DDX5 protein expression was detected by Western blotting using anti-DDX5 antibodies, and FMDV structural proteins were detected using anti-FMDV serum. (**C**) The relative FMDV RNA levels were analyzed by RT-qPCR at indicated time points. (**D**) Viral progeny yields were examined based on TCID_50_ analysis. (**E**) PK-15 cells were transfected with Flag-DDX5 and empty vector (EV) for 24 h, followed by FMDV infection. Cell lysates were harvested at indicated time points and subjected to Western blot analysis. (**F**) The relative FMDV RNA levels were analyzed by RT-qPCR at indicated time points. (**G**) Viral progeny yields were examined based on TCID_50_ analysis. (**H**) Schematic of full-length DDX5 and its truncated mutants. (**I**) Full-length DDX5 and its truncated mutants were transfected into PK-15 cells, and then infected with FMDV at an MOI of 1. Cell lysates were harvested at indicated time points and subjected to Western blot analysis. Statistical significance was assessed based on the *P*-value; *P < 0.05, **P < 0.01, and ***P < 0.001.

To further assess the role of DDX5 in FMDV proliferation, PK-15 cells were edited using CRISPR-Cas9 to generate DDX5-KO cells. The DDX5-KO cell line was confirmed by sequencing, showing two nucleotide deletions in the DDX5 gene (Fig. 2A). Notably, the knockout of DDX5 did not obviously affect the cell growth rate of DDX5-KO cell lines compared to wild-type cells (data not shown). These cells, referred to as DDX5-KO, were then infected with FMDV in parallel with the parental cell line to detect the effect of DDX5-KO on FMDV replication. As shown in Fig. 2B, the expression of viral proteins VP0, VP1-VP2, and VP3 was significantly enhanced in the DDX5-KO cells compared to wild-type cells at 3, 5, 7, and 9 hp.i. Consistent with the result, elevated FMDV RNA and progeny virus yields were also observed in the DDX5-KO cells (Fig. 2C and D). In contrast, overexpression of DDX5 in the PK-15 cell line significantly suppressed FMDV replication (Fig. 2E through G). These results confirm that DDX5 functions as a negative regulator of FMDV proliferation.

DDX5 consists of three functional domains: a DEAD core region (DEADc) in the N-terminal domain, a Helicase C-terminal domain (HELICc), and P68HR (Fig. 2H). To identify which functional domain of DDX5 is responsible for this regulatory role, PK-15 cells were transfected with plasmids expressing full-length and various truncated forms of DDX5 fused with a Flag tag for 24 h, and then infected with FMDV. As shown in Fig. 2I, only the full-length DDX5 retained the ability to inhibit FMDV replication, while all truncated forms failed to exert this effect. These findings indicate that the full-length DDX5 is essential for its antiviral function.

### DDX5 inhibits the translation of FMDV

**Fig 3:**
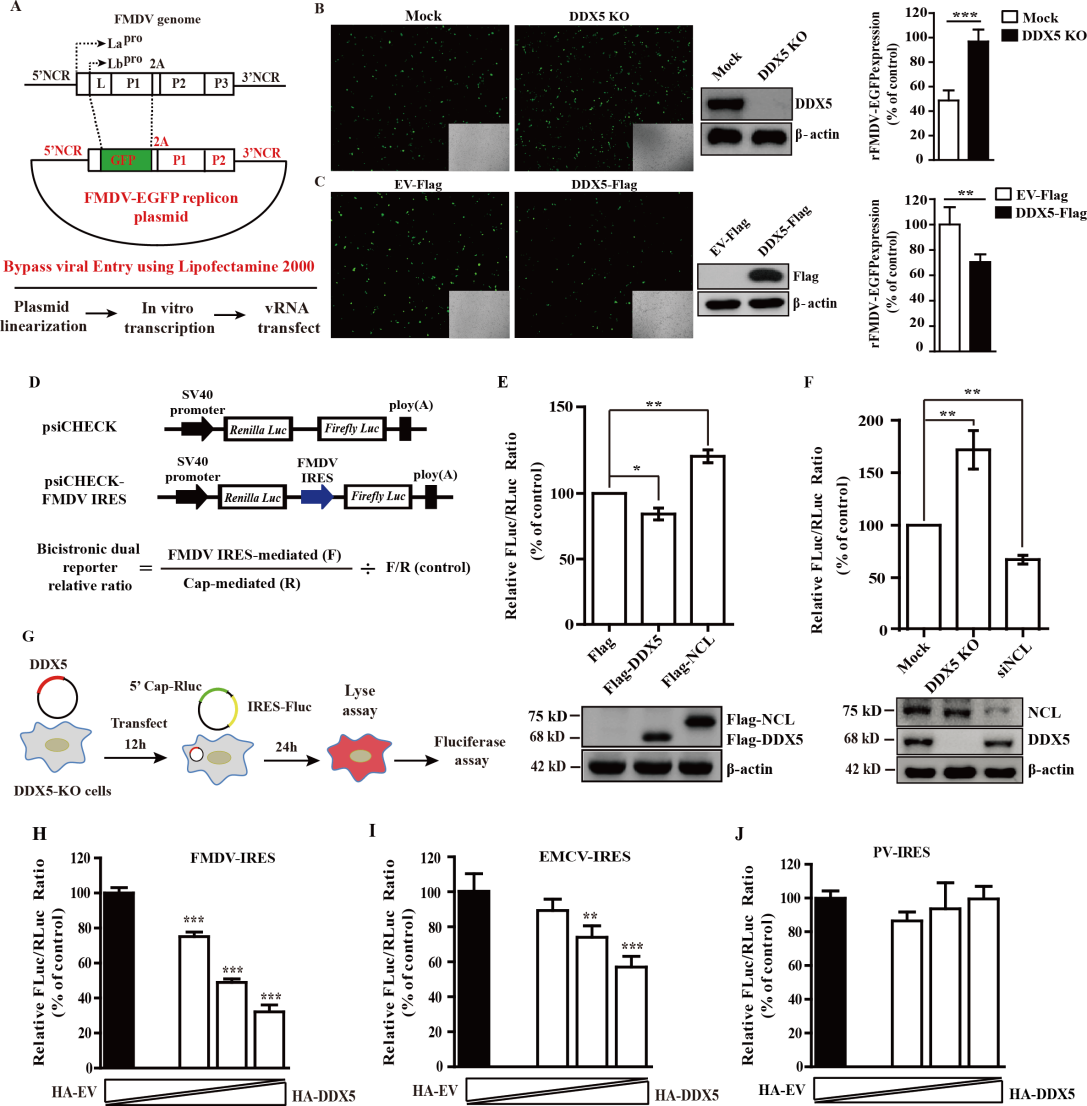
DDX5 negatively regulates FMDV IRES-driven translation. (**A**) Schematic illustration of the subgenomic rFMDV-EGFP replicon. (**B**) DDX5-KO PK-15 or WT PK-15 cells were transfected with the replicon rFMDV-EGFP. Twelve hours post-transfection, cells were subjected to fluorescence analysis by fluorescence microscope and flow cytometry. (**C**) PK-15 cells were transfected with the DDX5-Flag or EV-Flag. At 24 h post-transfection, cells were transfected with replicon rFMDV-EGFP. Twelve hours post-transfection, cells were subjected to fluorescence analysis by fluorescence microscope and flow cytometry. (**D**) Schematic illustration of bicistronic luciferase reporter plasmids. (**E and F**) DDX5 overexpression and DDX5-KO of PK-15 cells were transfected with the luciferase reporter plasmids psiCHECK or psiCHECK-FMDV. At 24 hpt, the signal intensities of FLuc and RLuc were detected. (**G**) Schematic diagrams of plasmid-based DDX5-rescued and dual-luciferase bicistronic reporter assays. (**H–J**) DDX5-rescued cells were transfected with different dual-luciferase bicistronic reporter plasmids containing FMDV-, EMCV-, and PV-IRES. At 24 hpt, the signal intensities of FLuc and RLuc were detected. Statistical significance was assessed based on the *P*-value; *P < 0.05, **P < 0.01, and ***P < 0.001.

Based on the above results, we proposed that DDX5 might participate in FMDV IRES-dependent translation or viral RNA synthesis. Therefore, we bypassed FMDV entry by directly transfecting cells with an *in vitro*-transcribed FMDV subgenomic replicon RNA (rFMDV-enhanced green fluorescent protein [EGFP]), in which the structural protein genes have been replaced with the EGFP reporter gene (Fig. 3A). As shown in Fig. 3B and C, the expression level of EGFP was significantly enhanced in DDX5-KO cells, whereas the replicon activity was obviously decreased when DDX5 was ectopically expressed, suggesting that DDX5 is implicated in the translation and/or replication of FMDV.

To determine the specific step of the FMDV life cycle influenced by DDX5, we examined its effect on FMDV translation using the plasmid-based dual-luciferase reporter system, psiCHECK-FMDV IRES. This system contains a cap-dependent *Renilla luciferase* (RLuc) gene and an FMDV IRES-driven *Firefly luciferase* (FLuc) gene (Fig. 3D). We found that the overexpression of DDX5 resulted in a significant reduction of FMDV IRES activity (Fig. 3E), while the knockout of DDX5 remarkably enhanced IRES activity (Fig. 3F). Meanwhile, we also found that the effect of DDX5 on FMDV IRES was opposite to the effect of NCL, which has been demonstrated as a positive ITAF for FMDV translation (Fig. 3E and F). Moreover, we also found that the overexpression or knockout of DDX5 did not influence the transcription or RNA stability of bicistronic reporter gene (data not shown). To further investigate the influence of DDX5 on IRES-mediated translation in picornaviruses, we conducted a rescue experiment by ectopically expressing full-length DDX5 in DDX5-KO cells in a dose-dependent manner, while maintaining equal DNA transfection levels by supplementing with HA-EV (empty vector). Subsequently, DDX5-rescued cells were transfected with different dual-luciferase bicistronic reporter plasmids containing FMDV-, EMCV-, PV-, CSFV-, and SVA-IRES (Fig. 3G). We then analyzed the activities of Rluc (Cap) and FLuc (IRES), relative to the negative control of HA-EV that was transfected alone. The result indicated that in DDX5-KO cells, the presence of DDX5 significantly inhibited FMDV and EMCV (type II) IRES-driven translation in a dose-dependent manner (Fig. 3H and I), but not of the type I IRES of PV (Fig. 3J), type III IRES of CSFV, and type IV IRES of SVA (data not shown). Collectively, these results indicate that DDX5 functions as a negative regulator of FMDV IRES-driven translation.

### DDX5 interacts with FMDV IRES and 3′UTR

**Fig 4:**
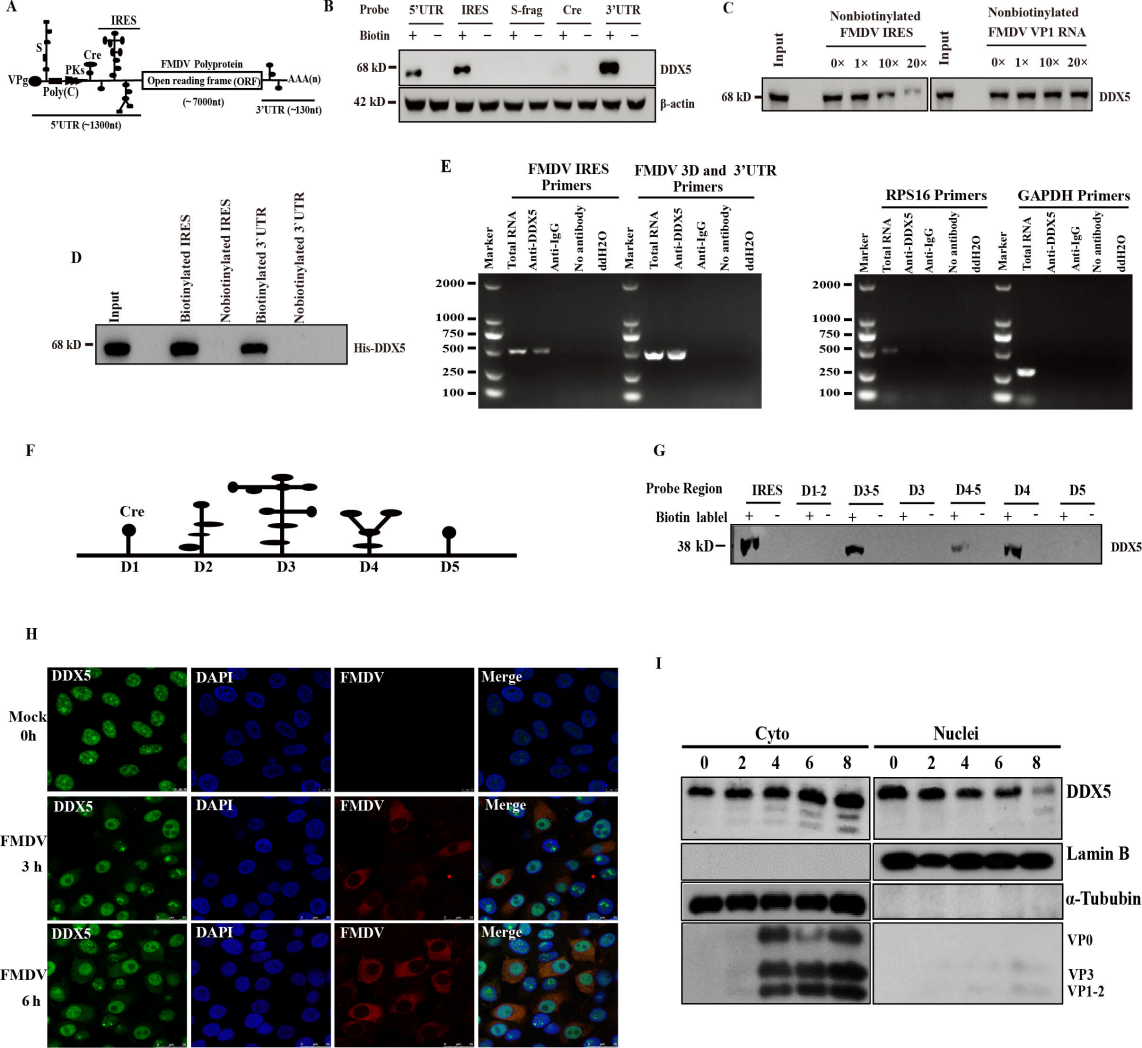
DDX5 interacts with FMDV IRES. (**A**) Schematic representation of the FMDV genome. (**B**) Cell lysates were incubated with the biotin-labeled 5′UTR, S-frag, Cre, IRES, and 3′UTR of FMDV, respectively. Non-biotinylated RNA probes were utilized as negative controls. These streptavidin beads were washed following the manufacturer’s instructions and subjected to immunoblot analysis with anti-DDX5 monoclonal antibody. (**C**) The different amounts of non-biotinylated FMDV IRES and VP1 RNA were added to compete with the biotinylated FMDV IRES that interacted with DDX5. (**D**) The biotinylated and non-biotinylated FMDV IRES and the purified recombinant 6×His DDX5 expressed in *E. coli* were performed in RNA-pulldown assay. (**E**) The FMDV-infected PK-15 cells were lysed and incubated with anti-DDX5 antibody for RNA immunoprecipitation. Negative controls included anti-IgG, no antibody, and ddH_2_O. RNA was extracted and amplified by RT-PCR using primers directed against FMDV IRES, 3D to 3′UTR, RPS16, and GAPDH. (**F**) Schematic representations of FMDV IRES truncations. Seven forms of IRES were IRES D1-5, IRES D1-2, IRES D3-5, IRES D3, IRES D4-5, IRES D4, and IRES D5. (**G**) The full-length IRES and its truncated forms were transcribed *in vitro* and biotinylated. PK-15 cell lysates were used to perform RNA-pulldown assay with biotinylated IRES and its truncations. (**H**) PK-15 cells were mock- or FMDV-infected and then fixed at 4 or 8 hp.i. The cells were permeabilized and then probed with indirect immunofluorescence for FMDV (red) and DDX5 (green). Nuclei are indicated by DAPI staining (blue). Cells were visualized by confocal microscopy. (**I**) Nuclear and cytoplasmic fractions were isolated from PK-15 cells at the indicated times post-FMDV infection and analyzed by Western blot using the indicated antibodies.

Our previous LC-ESI-MS/MS data showed that DDX5 interacts with the FMDV 5′- and 3′UTR. Meanwhile, we also found that DDX5 negatively regulates FMDV IRES-dependent translation. To further confirm the interaction between DDX5 and viral RNA, an RNA-protein pulldown assay was performed. Cellular extracts from PK-15 cells were incubated with biotinylated RNA probes, which included the 5′UTR, S-frag, Cre, IRES, and 3′UTR regions of the FMDV genome (Fig. 4A). Western blot analysis revealed that DDX5 interacted with FMDV 5′UTR, IRES, and 3′UTR, but not with the S-frag and Cre regions (Fig. 4B). Meanwhile, a competition assay was implemented using RNA-pulldown assay with different amounts of non-biotinylated IRES or FMDV VP1 RNA. The results revealed that the interaction between DDX5 and FMDV IRES was outcompeted by non-biotin-FMDV IRES rather than FMDV VP1 RNA, indicating that the interaction is FMDV IRES-specific (Fig. 4C). To investigate whether DDX5 directly binds to the FMDV IRES and 3′UTR, the recombinant 6×His-DDX5 expressed in *Escherichia coli* was purified and subjected to RNA-pulldown assay with biotinylated IRES and 3′UTR. The result showed that the biotinylated IRES and 3′UTR directly bind to the recombinant 6×His-DDX5, demonstrating that DDX5 directly interacts with FMDV IRES and 3′UTR (Fig. 4D).

To further explore the reciprocal interaction between DDX5 and viral RNA, we performed RNA co-immunoprecipitation assays. Cell lysates were incubated with a specific anti-DDX5 antibody and an isotype control anti-IgG at 5 hp.i. with FMDV (MOI = 1). Total RNAs were extracted from the immune complexes and amplified by RT-PCR using specific primers targeting FMDV IRES, FMDV 3D to 3′UTR, or RPS16 and GAPDH (control primers). Our results indicated that both the FMDV IRES and FMDV 3D to 3′UTR were efficiently amplified in the total RNA and anti-DDX5 samples, whereas neither RPS16 nor GAPDH was amplified in the anti-DDX5 immunoprecipitated samples (Fig. 4E), suggesting that DDX5 specifically interacts with the viral RNA in FMDV-infected cells.

It is known that FMDV IRES has five important domains: D1–D5. To clarify which domains play a significant role in binding DDX5, a series of truncated forms of FMDV IRES were synthesized by *in vitro* transcription and subsequently biotinylated to evaluate their binding ability with DDX5 through an RNA-pulldown assay (Fig. 4F). A Western blot analysis showed that except for full-length IRES, biotin-RNA probes D3–5, D4–5, and D4 were co-purified with DDX5, indicating that the D4 regions of FMDV may be mainly responsible for the binding of DDX5. Given that DDX5 is predominantly nuclear while FMDV translation and replication occur in the cytoplasm, we next examined whether infection triggers DDX5 nuclear‑cytoplasmic translocation. Laser confocal microscopy revealed that FMDV infection promoted the redistribution of DDX5 from the nucleus to the cytoplasm at 3 hp.i. and 6 hp.i. (Fig. 4G). Consistent with this observation, nuclear‑cytoplasmic fractionation assays demonstrated that DDX5 progressively accumulated in the cytoplasm from 4 hp.i. onward (Fig. 4I). Taken together, these results strongly indicate that DDX5 directly interacts with FMDV IRES and 3′UTR.

### DDX5 regulates the translation initiation of FMDV mRNA

**Fig 5:**
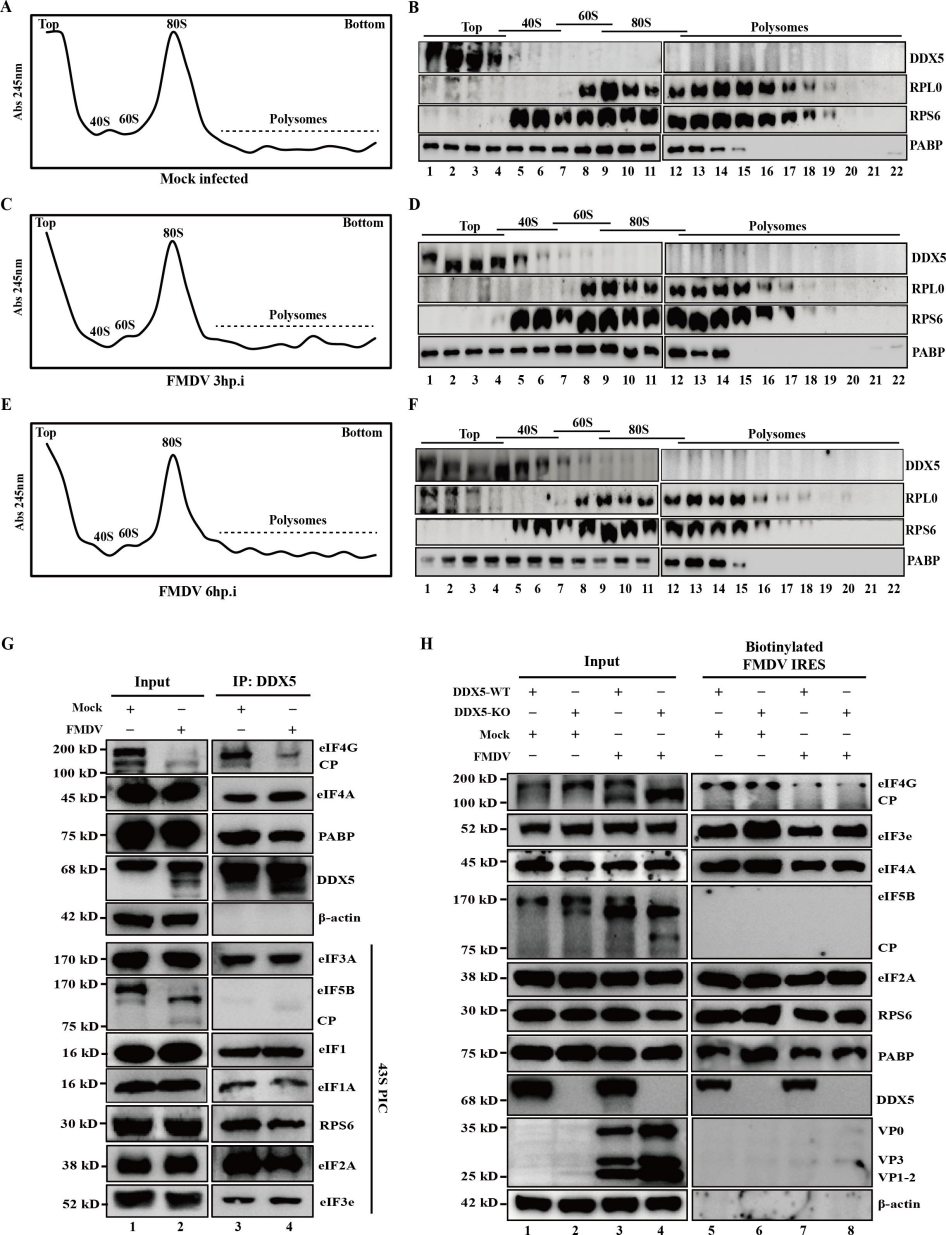
DDX5 regulates the translation initiation of FMDV mRNA. (**A–F**) Cellular extracts were prepared from mock-infected or FMDV-infected (MOI = 1) PK-15 cells at 3 and 6 hp.i. The extracts were resolved by sedimentation through 10%–50% sucrose gradients and subsequently fractionated. Polysome profiles (left) were generated by monitoring the OD_254_ across the gradient fractions. The collected fractions were then analyzed by Western blotting to assess the cosedimentation of DDX5 with ribosomal subunits (40S, 60S), monosomes (80S), or polysomes (right). (**G**) Lysates from mock-infected and FMDV-infected PK-15 cells (4 hp.i.) were subjected to co-immunoprecipitation with an anti-DDX5 antibody, and the precipitates were analyzed by Western blotting (lanes 3 and 4). (**H**) DDX5-WT and DDX5-KO cells were infected with FMDV at an MOI of 1. At 4 hp.i., cell lysates were harvested and subjected to an RNA-pulldown assay with biotinylated FMDV IRES. The proteins associated with translation initiation complexes were analyzed by Western blotting (lanes 5 to 8).

To investigate the association of DDX5 with ribosomal subunits, extracts from mock- or FMDV-infected PK-15 cells were subjected to ultracentrifugation to separate the ribosomal subunits (40S and 60S), monosomes (80S), and polysomes by sucrose gradient fractionation. The sedimentation profiles of ribosomal complexes were determined by measuring OD_254_ absorbance across the gradient fractions and visualizing the ribosomal protein distribution via immunoblotting (Fig. 5A through F). RPS6 and RPLP0 were employed as specific markers for the 40S and 60S subunits respectively, while PABP, which was almost present in all gradient fractions, was used as a negative control. Notably, absorbance profiles revealed a reduction in 80S monosome levels following FMDV infection, particularly at 6 hp.i. (Fig. 5A and E), indicating that FMDV infection damages cellular global translation. Compared with the mock-infected cells, DDX5 was primarily distributed to the top gradient fractions, while FMDV infection induced partial redistribution of DDX5 to 40S ribosomal subunit fractions (Fig. 5B through F), suggesting that DDX5 may associate with the translation initiation of FMDV mRNA.

FMDV shuts down cellular cap-dependent translation by cleaving key translation initiation factors eIF4G and eIF5B and recruits many eIFs (such as eIF2, eIF3, and eIF4) and ribosome to translate viral proteins via IRES (6, 12). Moreover, the above results have shown that DDX5 interacts with FMDV IRES and negatively regulates virus translation. Therefore, we speculated that DDX5 may modulate the formation of FMDV translation initiation complexes. To clarify the role of DDX5 in this process, cellular lysates from mock- and FMDV-infected PK-15 cells were subjected to immunoprecipitation using an anti-DDX5 monoclonal antibody to detect the proteins associated with translation initiation factors. Western blot analysis revealed that DDX5 co-precipitated with a range of host factors associated with translation initiation, including eIF4G, eIF4A, and PABP, as well as the components of 43S pre-initiation complex (43S PIC), but not with eIF5B (Fig. 5G). These results support the hypothesis that DDX5 interacts with key initiation factors to regulate FMDV IRES-mediated translation.

As translation initiation is a critical rate-limiting step encompassing 43S PIC assembly, mRNA recruitment, and 60S ribosomal subunit joining (8), we further examined whether DDX5 is directly involved in FMDV IRES-mediated translation initiation using a biotin-labeled RNA-pulldown assay. As depicted in Fig. 5H, the biotinylated FMDV IRES efficiently pulled down several initiation factors, including eIF2α, eIF3e, eIF4A, eIF4G, PABP, and RPS6, but not eIF5B (lanes 5 to 8). Notably, DDX5 depletion did not significantly disrupt the binding between the FMDV IRES and these initiation factors, indicating that DDX5 is not essential for the initial recruitment of the IRES-mediated translation initiation complex. Collectively, these results suggest that DDX5 does not serve as a core component for FMDV IRES-driven translation but functions as a regulatory factor. Thus, we propose that DDX5 may modulate a subsequent step, potentially the joining of the 60S ribosomal subunit, to ultimately interfere with the formation of 80S ribosomes.

### DDX5 interferes with 80S ribosome assembly on FMDV IRES

**Fig 6:**
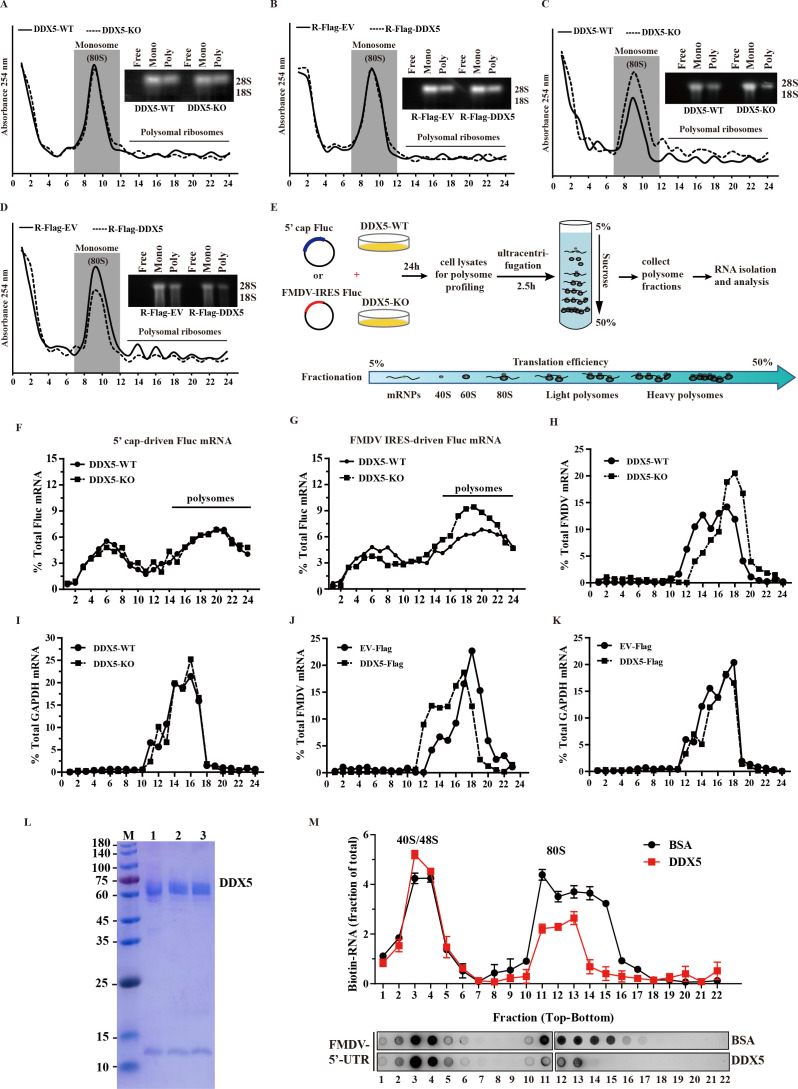
DDX5 interferes with 80S ribosome assembly on FMDV IRES. (**A and B**) DDX5-KO and -overexpression of PK-15 cells were incubated with CHX for 15 min and then the protein lysates were collected or (**C and D**) DDX5-KO and -overexpression of PK-15 cells were infected at 8 hp.i. Cells were incubated with CHX for 15 min and then the protein lysates were collected. (A to D) Lysates were loaded on 5 to 50% sucrose and ultra-centrifuged at 36,000 rpm for 3 h. Polysome profiles were generated through the measurement of optical density at 254 nanometers (OD_254_) within the gradient fractions. Polysome profile curves represent the mean absorbances from two independent experiments. Aliquots were collected from free mRNA, monosome, or polysome fractions and separated by gel electrophoresis. (**E**) Schematic diagrams of polysome profiling for 5′ cap-FLuc or FMDV IRES FLuc in DDX5-KO or WT PK-15 cells. (**F**) The distribution of 5′ cap-driven FLuc mRNA among different polysome fractions. (**G**) The distribution of FMDV IRES-driven FLuc mRNA among different polysome fractions. (**H and I**) DDX5-KO cells were infected with FMDV for 6 h and treated with CHX for 15 min. Lysates were subjected to ultracentrifugation at 36,000 rpm for 3 h. These obtained fractions were treated with TRIzol for RNA extraction. Total RNA was converted to cDNA and subjected to RT-qPCR for the amplification of the FMDV and GAPDH. (**J and K**) PK-15 cells were transfected with EV-Flag or DDX5-Flag for 24 h. The overexpressed PK-15 cells were infected with FMDV for 6 h and treated with CHX for 15 min. Lysates were subjected to ultracentrifugation at 36,000 rpm for 3 h. Fractions were treated with TRIzol for RNA extraction. Total RNA was converted to cDNA and subjected to RT-qPCR for the amplification of the FMDV and GAPDH. (**L**) SDS-PAGE analysis of purified recombinant DDX5 protein. (**M**) Biotinylated FMDV 5′UTR RNA was incubated with recombinant DDX5 protein or a BSA control in ribosome assembly buffer at 30°C for 40 min. Following incubation, the complexes were resolved by sucrose density gradient ultracentrifugation. The distribution of the biotinylated RNA across the gradient fractions was detected by dot blotting using streptavidin-HRP and quantified by densitometry.

Considering that DDX5 interacts with IRES-driven translation initiation factors and reduces IRES activity without affecting the initial recruitment of the IRES-mediated translation initiation complex, we speculate that DDX5 may block the assembly of the 80S ribosome by interfering with the recruitment of the 60S ribosomal subunit to the 43S PIC. This interference would lead to reduced ribosomal occupancy on viral RNA and consequent repression of FMDV translation. To mechanistically elucidate the role of DDX5 in translational regulation, we employed polysome profiling to separate ribosomal complexes by sedimentation velocity, enabling quantitative assessment of translational efficiency and integrity of ribosomal assembly. As shown in Fig. 6A and B, neither overexpression nor knockout of DDX5 significantly altered the polysome profiles in uninfected cells. In contrast, upon FMDV infection, DDX5-KO cells exhibited an obvious increase in the abundance of both monosomes and polysomes compared to wild-type controls (Fig. 6C), whereas ectopic DDX5 expression reduced their abundance compared to the control group (Fig. 6D). Subsequently, DDX5-KO and -WT cells were transfected with equimolar amounts of cap- or IRES-driven FLuc mRNA to explore the effect of DDX5 on ribosomal occupancy, respectively (Fig. 6E). As expected, DDX5 did not change the overall distribution of 5′ cap-driven FLuc mRNA among different polysome fractions (Fig. 6F). However, FMDV IRES-driven FLuc mRNA amount was found to be significantly increased in DDX5-KO cells compared with DDX5-WT (Fig. 6G). To further determine whether DDX5 inhibits IRES-driven FMDV mRNA amount during viral infections, we compared ribosome abundance on FMDV mRNA with cellular capped GAPDH mRNA in DDX5-WT and DDX5-KO cells. The lysates obtained from FMDV-infected DDX5-WT and DDX5-KO cells were fractionated by sucrose density gradient centrifugation, and each fraction with specific mRNA was determined through a RT-qPCR analysis. We observed that the levels of FMDV mRNA peaked in fraction 16 of the DDX5-WT cells, whereas in DDX5-KO cells, the distribution of FMDV mRNA shifted toward heavier fractions, peaking in fraction 18 (Fig. 6H). Furthermore, in FMDV-infected cells ectopically expressing DDX5, the distribution of FMDV shifted toward lighter fractions compared to the empty vector (EV-Flag) control group (Fig. 6J). The distribution of cap-driven GAPDH mRNA, however, was not affected in both FMDV-infected DDX5-WT and DDX5-KO cells (Fig. 6I), as well as in FMDV-infected DDX5-Flag and EV-Flag cells (Fig. 6K), indicating that DDX5 affects the ribosome abundance on FMDV mRNA.

To further elucidate the mechanism that DDX5 reduces ribosome occupancy on viral RNA, we examined its effect on ribosome assembly using a rabbit reticulocyte lysate (RRL) system. The biotin-labeled FMDV IRES RNA was incubated with RRL in the presence of either recombinant DDX5 protein (Fig. 6L) or a BSA control. The assembly mixtures were then separated by sucrose density gradient ultracentrifugation, and ribosomal complexes were analyzed via dot blotting. As expected, the 80S ribosome assembly peak was detected after 40 min of incubation in the BSA control, whereas this peak was substantially diminished in reactions containing DDX5 (Fig. 6M). Taken together, these results indicate that DDX5 inhibits FMDV IRES activity by blocking the formation of the 80S ribosome.

### DDX5 inhibits viral RNA synthesis via interacting with FMDV 3D^pol^

**Fig 7:**
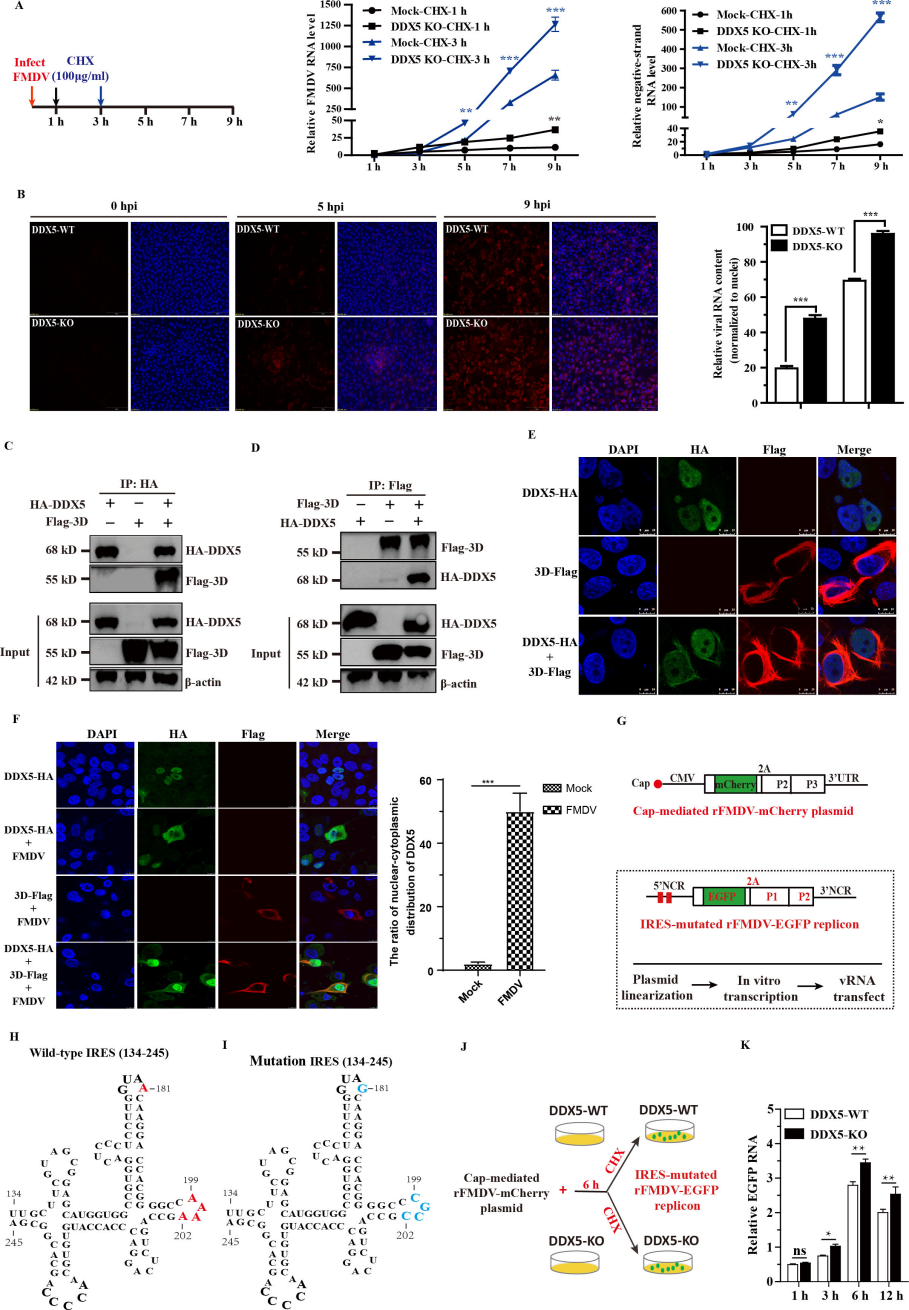
DDX5 inhibits viral RNA synthesis via interacting with FMDV 3D^pol^. (**A**) DDX5-KO cells were infected with FMDV, and CHX was added to the culture medium at 1 or 3 hp.i. RNA was extracted at the indicated time, and the levels of positive-strand and negative-strand RNA were determined by RT-qPCR. (**B**) DDX5-WT and DDX5-KO cells were infected with FMDV (MOI of 1). Cells were used for viral RNA FISH (SweAMI FISH) assay. The hybridization signals were detected using a fluorescence microscope (OLYMPUS, IX73, Japan) and analyzed using Caseviewer 2.4. (**C and D**) PK-15 cells were co-transfected with HA-DDX5 and Flag-3D^pol^. Cell lysates were immunoprecipitated with anti-HA or anti-Flag and then analyzed by Western blotting. (**E**) PK-15 cells were transfected with HA-DDX5 and Flag-DDX5 for 24 h. Cells were fixed with 4% PFA and stained with antibodies against HA (green) and Flag (red), nuclei were stained with DAPI (blue). The samples were observed under a confocal microscope. (**F**) PK-15 cells were transfected with HA-DDX5 and Flag-DDX5, followed by FMDV infection at directed time points. Cells were fixed with 4% PFA and stained with antibodies against HA (green) and Flag (red), nuclei were stained with DAPI (blue). The samples were observed under a confocal microscope. (**G**) Schematic diagram of the cap-driven rFMDV-mCherry plasmid and the IRES-mutated rFMDV-EGFP replicon. (**H**) The D3 region of WT-IRES (134-245), GNRA, and RAAA motifs is shown in red. (**I**) The mutations of the GNRA and RAAA motifs are shown in blue. (**J**) Schematic diagrams of co-transfection of the cap-driven rFMDV-mCherry plasmid and the IRES-mutated rFMDV-EGFP replicon in DDX5-WT and DDX5-KO cells. (**K**) RNA was extracted at the indicated time, and the levels of EGFP RNA were determined by RT-qPCR. Statistical significance was assessed based on the *P*-value; *P < 0.05, **P < 0.01, and ***P < 0.001.

The above results indicate that DDX5 interacts with FMDV IRES and 3′UTR, respectively (Fig. 4). Therefore, we speculate that DDX5 may also be involved in FMDV RNA synthesis. To investigate the effect of DDX5 on viral RNA synthesis, the CHX (a protein synthesis inhibitor) was used to separate the translation and viral RNA synthesis processes. When CHX was added to inhibit virus translation at 1 or 3 hp.i., the viral RNA levels were significantly enhanced in DDX5-KO cells, particularly at 3 hp.i. (Fig. 7A). This finding suggests that DDX5 also negatively regulates FMDV replication by inhibiting RNA synthesis. To confirm this result, we performed viral RNA fluorescence *in situ* hybridization (SweAMI FISH) assay to detect FMDV RNA levels in FMDV-infected DDX5-WT and DDX5-KO cells. As shown in Fig. 7B, when viral translation was blocked at 3 hp.i., viral RNA levels were significantly higher in DDX5-KO cells than DDX5-WT, indicating that DDX5 represses viral RNA synthesis.

The RNA-dependent RNA polymerase 3D^pol^ of FMDV is an essential component of viral RNA replication complexes and mainly responsible for the synthesis of viral RNA. To further elucidate the mechanism by which DDX5 reduces viral RNA synthesis, we examined the association of DDX5 with 3D^pol^ by immunoblot assay. The immunoprecipitation data showed that DDX5 physically interacted with 3D^pol^ in PK-15 cells co-transfected with HA-DDX5 and Flag-3D^pol^ (Fig. 7C and D). Meanwhile, the localization of HA-DDX5 with Flag-3D^pol^ was performed by immunofluorescence analysis. As expected, our results indicated that HA-DDX5 localized in the cell nucleus, but it was translocated into the cytoplasm when Flag-3D^pol^ was co-expressed with HA-DDX5 (Fig. 7E). To further explore the interaction of DDX5 with 3D^pol^ in FMDV-infected cells, HA-DDX5 and Flag-3D^pol^ were co-transfected into PK-15 cells for 24 h and infected with FMDV. As shown in Fig. 7F, FMDV infection significantly enhanced the translocation of HA-DDX5 to the cytoplasm and co-localized with Flag-3D^pol^. Therefore, we speculate that the interaction between DDX5 and FMDV 3D^pol^ may inhibit viral RNA synthesis.

During FMDV infection, the viral genomic RNA serves as the template for translation and RNA replication, thereby tightly coupling these two processes. To dissociate FMDV RNA synthesis from translation and eliminate potential confounding effects of DDX5 on IRES-mediated translation, we constructed a cap-driven rFMDV-mCherry plasmid that is translationally competent but replication-deficient (Fig. 7G, upper), and an IRES-mutated rFMDV-EGFP replicon. The replicon maintains replication capacity when supplied with 3D^pol^ expressed *in trans* from the cap-driven rFMDV-mCherry plasmid, but is defective in IRES-driven translation (Fig. 7G, lower). Consistent with previous reports (22, 23), we confirmed that the GUAA and AAAA conserved motifs within domain 3 of the FMDV IRES are essential for its function. Substitutions of A181 to G and A199AAA202 to CGCC completely abrogated IRES-mediated translation activity (Fig. 7H and I and S1). To specifically assess the role of DDX5 in viral RNA replication independent of translational effects, we transfected the cap-driven rFMDV-mCherry plasmid into both DDX5-WT and DDX5-KO cells. As expected, DDX5-KO did not affect translation of the cap-driven rFMDV-mCherry plasmid (Fig. S2). At 6 h post-transfection, CHX was added to block new protein synthesis, followed by transfection with the IRES-mutated rFMDV-EGFP replicon RNA (Fig. 7J). Subsequent quantification revealed that DDX5-KO significantly enhanced rFMDV-EGFP replicon RNA accumulation compared to DDX5-WT cells (Fig. 7K), indicating that DDX5 directly restricts viral RNA synthesis through its interaction with 3D^pol^.

### FMDV antagonizes DDX5-mediated repression via 3ABCD protease precursor

**Fig 8:**
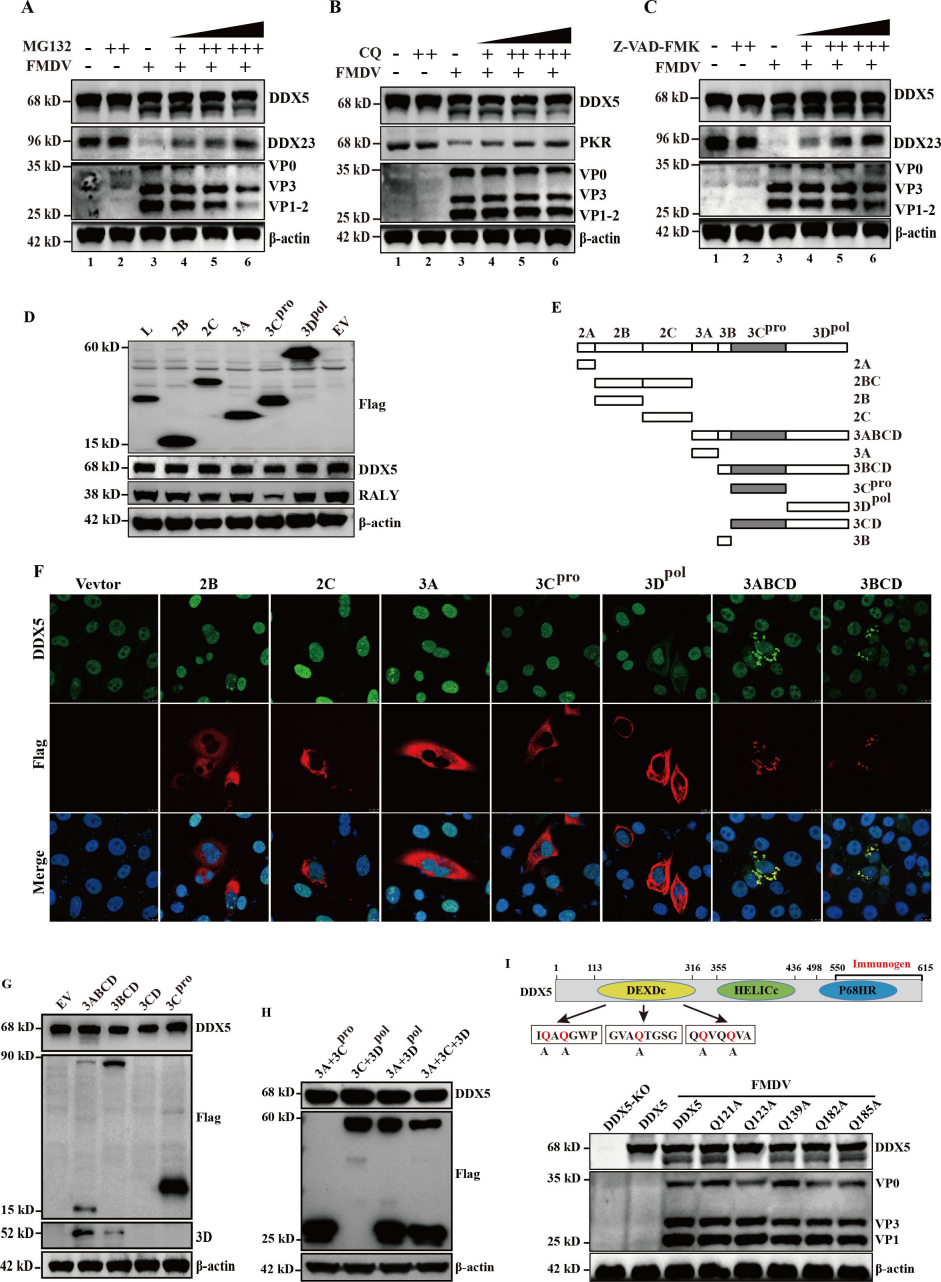
FMDV antagonizes DDX5-mediated repression. (**A–C**) PK-15 cells were infected with and treated with proteasome inhibitor (MG132: 2, 20, or 40 µM), lysosomal pathway inhibitor (CQ: 20, 50, or 70 µM), and caspase general inhibitor (Z-VAD-FMK: 10, 50, or 70 µM), and samples were collected at 9 hp.i. Samples were analyzed through Western blotting. (**D**) PK-15 cells were transfected with FMDV flag-tagged non-structure proteins, L^pro^, 2B, 2C, 3A, 3C^pro^, and 3D^pol^. At 24 h post-transfection, samples were collected and analyzed via Western blotting. (**E**) Schematic showing FMDV non-structural proteins and processing intermediates. (**F**) PK-15 cells were transfected with plasmids expressing FMDV non-structural proteins and intermediates. The cells were fixed and stained with antibodies against DDX5 (green) and Flag (red), nuclei were stained with DAPI (blue). The samples were observed under a confocal microscope. (**G**) PK-15 cells were transfected with Flag-3ABCD, -3B.C.BCD, -3CD, and -3C for 24 h. The cells were harvested and subjected to Western blot analysis. (**H**) PK-15 cells were co-transfected with Flag-3A and -3C, Flag-3C and -3D, Flag-3A and -3D, or Flag-3A, -3C, and -3D for 24 h. The cells were harvested and subjected to Western blot analysis. (**I**) Potential 3Cpro cleavage site between aa 120 and 190 in DDX5. The regions that contain glutamine-to-alanine mutations in DDX5 are highlighted in red. DDX5-KO cells were transfected with these plasmids for 24 h, infected with FMDV, and then harvested for Western blot analysis.

Our data indicated that DDX5 negatively regulates FMDV replication by repressing IRES-mediated translation activity and viral RNA synthesis. However, the expression of DDX5 was significantly reduced in the late stage of FMDV infection (Fig. 1C). Therefore, we speculate that DDX5 is counteracted after FMDV infection. To determine whether FMDV antagonizes the restriction of DDX5 via canonical degradation pathways, we inhibited the proteasome (MG-132), lysosome (CQ), and caspase pathways (Z-VAD(OMe)-FMK). The efficacy of each inhibitor was verified using established pathway substrates (DDX23 for proteasome/caspase; PKR for lysosome; Fig. 8A through C). However, we found that blocking the proteasome, lysosome, or caspase pathways failed to restore DDX5 levels upon infection. Notably, the partial recovery of DDX5 observed at high inhibitor concentrations could be attributed to the suppression of viral replication rather than direct pathway inhibition. (Fig. 8A through C, lanes 6). To further clarify how FMDV counteracts DDX5 protein, the plasmids encoding FMDV non-structural protein (Flag-L^pro^, -2B, -2C, -3A, -3C^pro^, and -3D^pol^) were transfected into PK-15 cells, respectively. The result showed that ectopic expression of viral proteins did not result in a reduction of DDX5 compared with the EV-Flag control (Fig. 7D). Conversely, ectopic expression of 3C^pro^ obviously degraded RALY, which is consistent with previous reports (1). Collectively, these results indicate that FMDV counteracts DDX5 through a mechanism distinct from canonical proteasome, lysosome, or caspase pathways, and one that is not mediated by any single viral non-structural protein alone.

The primary cleavage of the FMDV polyprotein is mainly processed by L^pro^ and 3C^pro^, producing 12 mature viral proteins and several intermediate precursors (Fig. 8E). Therefore, we suspect that several intermediate precursors may be involved in the degradation of DDX5. To confirm this hypothesis, non-structural proteins and several intermediate precursors with Flag were transfected into PK-15 cells and subjected to immunostaining. As shown in Fig. 8F, DDX5 co-localizes with non-structural protein 3D^pol^ and intermediate precursors 3ABCD and 3BCD. Considering that 3D^pol^ does not have protease activity, we transfected several intermediate precursors to investigate their effect on DDX5. As expected, we found that DDX5 was cleaved when 3ABCD was ectopically expressed, whereas 3BCD had no effect on the expression of DDX5 (Fig. 8G). Surprisingly, 3CD could not be detected for inscrutable reasons. To further investigate whether the mixed expression of 3A, 3C^pro^, and 3D^pol^ results in cleavage of DDX5, the relevant plasmids were co-transfected into PK-15 cells. As shown in Fig. 8H, the co-expression of these plasmids did not cause cleavage of DDX5, suggesting that 3ABCD was expressed *in cis* with 3C^pro^ to cause cleavage through an unknown process. Taken together, these results indicate that DDX5-mediated repression is antagonized by 3ABCD protease precursor.

To further identify the cleavage site in DDX5, we first analyzed patterns of cleaved DDX5 in FMDV-infected PK-15 cell lysates by immunoblot using a C-terminal-specific DDX5 antibody (Ab-C). As shown in Fig. 8I (top panel), this antibody detected a putative cleavage fragment of approximately 55 kDa. Given that FMDV 3Cpro preferentially cleaves after glutamine residues (24, 25), and considering the immunogen of DDX5 antibody and size of the cleavage fragment observed, we predicted that 3Cpro likely cuts at a glutamine residue located between aa 120 and 190 of DDX5. To determine the exact cleavage site of DDX5 by FMDV, we introduced point mutations (glutamine to alanine) at predicted residues within DDX5. These mutations are expected to prevent cleavage by FMDV 3C protease. We transfected these mutant plasmids (pcDNA3.1-DDX5) into DDX5-KO cells. After 24 h post-transfection, the cells were infected with FMDV, and the lysates were analyzed by immunoblotting. As shown in Fig. 8I (bottom panel), DDX5-Q123A was completely resistant to FMDV-mediated cleavage, indicating that the Gln-123 residue of DDX5 is likely the primary cleavage site for FMDV. To further investigate whether the individual cleavage fragments of DDX5 retain antiviral activity, we separately expressed the N- or C-terminal fragments in cells and then challenged them with FMDV infection. The result indicates that expression of individual N- or C-terminal fragments failed to restore antiviral activity against FMDV infection (Fig. S3), demonstrating that cleavage at Gln-123 by viral protease functionally disrupts the restrictive function of full-length DDX5.

### DDX5-depleted suckling mice are more susceptible to FMDV infection

**Fig 9:**
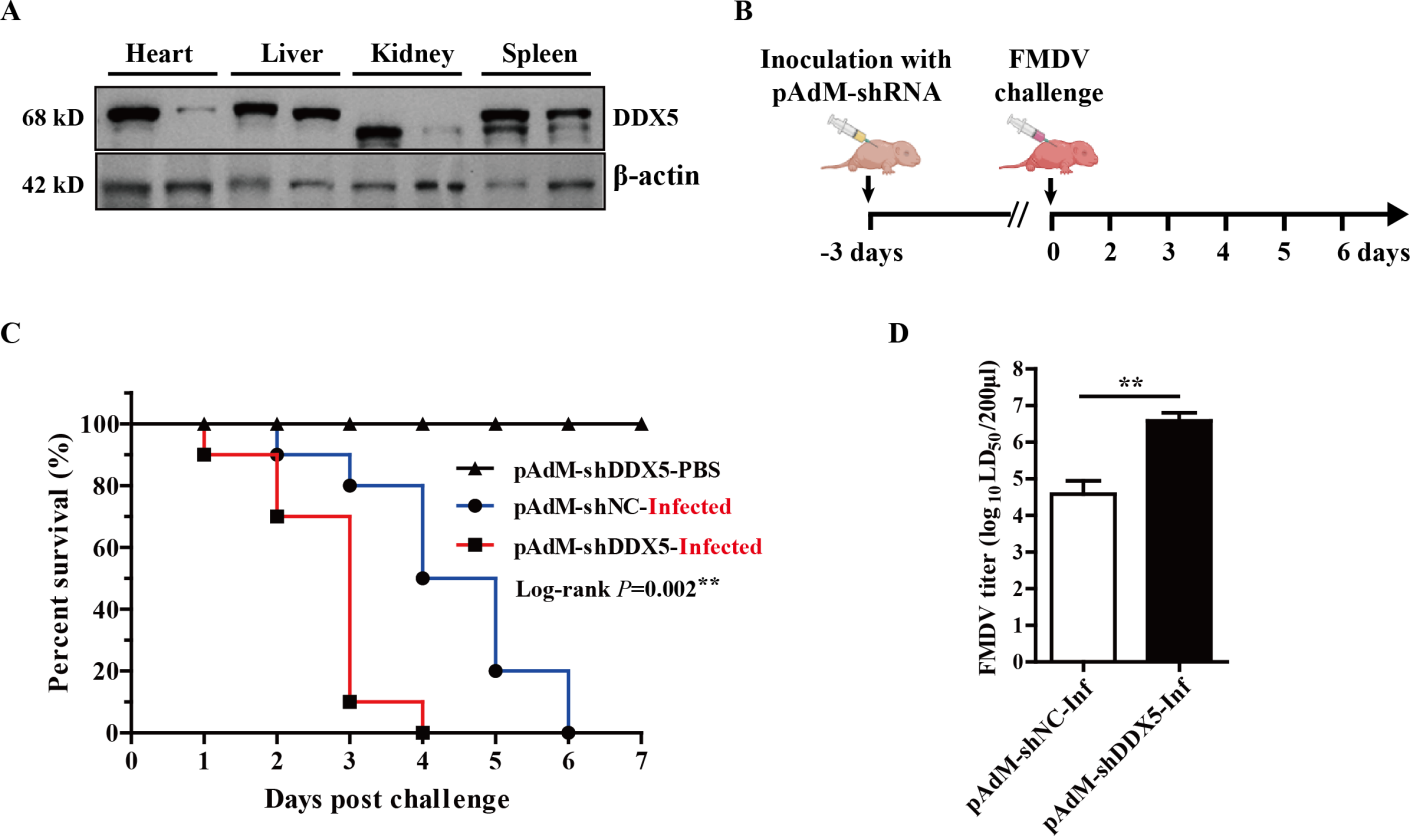
DDX5 knockdown decreases the survival of suckling mice. (**A**) Three-day-old suckling mice were inoculated through subcutaneous administration of pAdM-shNC or pAdM-shDDX5 at 1.0 × 10^8^ PFU. The mice were euthanized at 72 h post-inoculation, and their heart, liver, kidney, and spleen were collected and homogenized to assess the DDX5 protein expression. (**B**) Schematic illustration of FMDV infection procedure. At 72 h post-inoculation, mice were infected with 20 LD_50_ of FMDV O. (**C**) Mice survival rate was monitored for 7 days. (**D**) The muscle tissues were collected and TCID_50_ was performed. Statistical significance was assessed based on the *P*-value; *P < 0.05, **P < 0.01, and ***P < 0.001.

To elucidate the functional significance of DDX5 in a suckling mice model of FMDV infection *in vivo*, we generated DDX5-depleted neonatal mice through the subcutaneous administration of pAdM-shDDX5, an adenoviral vector designed to express three specific short hairpin RNAs (shRNAs) targeting DDX5. Western blot analysis showed that the expression of DDX5 protein was significantly downregulated in multiple tissues of the pAdM-shDDX5 treatment group compared with the control group treated with pAdM-shNC at 72 h post-inoculation (Fig. 9A). Subsequently, suckling mice treated with pAdM-shRNAs were exposed to an FMDV challenge via an inoculation of 20 times the median lethal dose (20×LD_50_). The post-infection survival trajectory of these mice was rigorously monitored for 7 days (Fig. 9B). A 100% mortality rate was found within 4 days post-infection in pAdM-shDDX5, while the control group-pAdM-shNC experienced complete mortality within a 6 day period post-infection (Fig. 9C). Consistent with these findings, a quantitative analysis of the viral load in the muscular tissue of the DDX5-depleted subjects showed a significant increase. Specifically, viral titers were approximately 10^2.1^-fold higher than the control group (Fig. 9D). These observations indicate that DDX5 depletion enhances the sensitivity of suckling mice to FMDV.

## DISCUSSION

**Fig 10:**
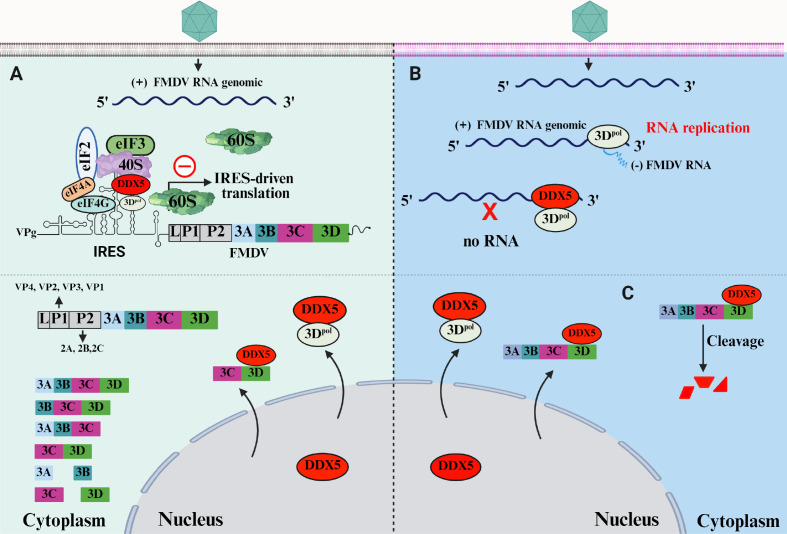
Schematic diagram illustrates the multifaceted role of DDX5 during FMDV infection. DDX5 is translocated into the cytoplasm from the nucleus by the interaction with 3D^pol^. (**A**) Within the cytoplasm, DDX5 interacts with the FMDV IRES to inhibit IRES-dependent viral translation by blocking the assembly of 80S ribosome. (**B**) DDX5 inhibits viral RNA synthesis via interfering with the interaction between 3D^pol^ and 3′UTR. (**C**) During the course of FMDV infection, DDX5-mediated inhibition is antagonized by 3ABCD protease precursor.

Picornaviruses are completely dependent on the host cell’s translation machinery for synthesizing their proteins, while concomitantly achieving the selective prioritization of viral mRNA translation by disrupting key steps of cap-dependent translation (26, 27). Accumulating evidence has revealed that picornaviruses utilize IRES to directly interact with cellular translational components to subvert host protein synthesis machinery (28–30). However, the precise mechanism by which IRES facilitates these interactions and manipulates the host’s translation machinery remains largely unclear. In this study, we identified DDX5 as a novel host restriction factor that negatively regulates FMDV translation and replication via a two-pronged strategy (Fig. 10). First, DDX5 impedes FMDV IRES activity by binding to the 5′UTR of FMDV genomic RNA, thereby disrupting the formation of the 80S ribosome and IRES-driven FMDV mRNA translation initiation. Second, we found that DDX5 also inhibits viral RNA synthesis via interacting with FMDV 3D^pol^. However, DDX5-mediated inhibition is counteracted by the FMDV 3ABCD protease precursor, suggesting a potential role in antiviral defense mechanisms.

DExH/D-box RNA helicases, constituting a highly conserved superfamily of RNA-binding proteins, have been demonstrated to modulate the unwinding of intermediate RNA duplexes and participate in RNA metabolism, including mRNA splicing, export, ribosome biogenesis, translation, and RNA decay (31–35). Intriguingly, these RNA helicases also play multifaceted roles in the life cycles of various viruses (35). Some function as viral sensors, such as DDX3, DDX41, and DHX9, while others play crucial roles in activating the innate immune response, including DDX60, DDX60L, and DDX23 (31, 36). Additionally, there are proteins that act as negative regulators and restrict the production of interferon (IFN) during viral infections (37). DDX5, a multifunctional RNA DEAD-box helicase, has been documented as a helper for viral replication (18, 34). A previous study reported that DDX5 interacts with PP2A-C β to deactivate IRF3, thereby inhibiting the production of IFN-I in cells infected with VSV (38). Furthermore, it was found that DDX5 facilitates viral infection via regulating N6-methyladenosine (m6A) levels of the DHX58 and NF-κB transcripts to hamper antiviral innate immunity during VSV infection (21). Recently, DDX5 was also identified as a positive regulator of JEV replication through interaction with the viral 3′UTR (20). However, our findings demonstrate that DDX5 acts as a negative regulator of FMDV propagation by modulating viral translation and RNA replication (Fig. 1 and 2).

FMDV translation is driven primarily by the highly structured IRES and 80S ribosome assembly, facilitated by a series of translation initiation factors and various auxiliary host factor ITAFs (6, 8). Here, DDX5 was identified as an ITAF that negatively regulates FMDV IRES-mediated translation (Fig. 3). Furthermore, we also further demonstrated that DDX5 interacts with the D4 regions of FMDV IRES (Fig. 4). Translation initiation is a critical, rate-limiting process involving multiple steps, including 43S PIC assembly, mRNA recruitment, and 60S ribosomal subunit joining (8). Immunoprecipitation and biotin-labeled RNA-pulldown assays revealed that DDX5 is not essential for the initial recruitment of the IRES-mediated translation initiation complex (Fig. 5). Considering that DDX5 reduces FMDV IRES-driven translation through IRES, we hypothesize that DDX5 may influence a subsequent step, potentially the joining of the 60S ribosomal subunit, to ultimately interfere with the formation of 80S ribosomes. Further research results indicated that the knockout of DDX5 promotes ribosome assembly (Fig. 6C), while ectopic DDX5 expression reduces monosome and polysome formation and abundance (Fig. 6D), suggesting that DDX5 may disrupt ribosome assembly via its interaction with the IRES. To further confirm this speculation, we examined the effect of DDX5 on ribosome assembly using RRL. Our results indicate that DDX5 inhibits FMDV IRES activity by blocking the formation of the 80S ribosome.

Besides affecting the FMDV IRES-driven translation, DDX5 may also influence viral RNA synthesis via interacting with the 3′UTR (Fig. 3). To further confirm this possibility, the protein synthesis inhibitor CHX was employed to distinguish between the processes of translation and RNA replication. Our findings demonstrated that DDX5 negatively regulates FMDV RNA synthesis (Fig. 6A). Meanwhile, the FISH assay also supported this hypothesis (Fig. 6B). FMDV 3D^pol^, an RNA-dependent RNA polymerase, plays an indispensable role in FMDV replication (39, 40). Therefore, we speculate that DDX5 interacts with FMDV 3Dpol, and our immunoprecipitation and confocal microscopy results confirm this interaction. The co-localization of DDX5 and 3Dpol is particularly enhanced upon viral infection, supporting the hypothesis that the interaction between DDX5 and FMDV 3D^pol^ inhibits viral RNA synthesis. However, a key challenge is that CHX treatment confounds translation and RNA synthesis, making it difficult to determine if subsequent RNA-level effects are due to viral protein level fluctuations or a direct role of DDX5 with 3Dpol. To unequivocally discriminate between these processes, we engineered a cap-driven rFMDV-mCherry plasmid and an IRES-mutated replicon (Fig. 6G through 6I). The results indicated that DDX5 directly restricts viral RNA synthesis through its interaction with 3Dpol (Fig. 6K).

FMDV shuts down cellular cap-dependent translation mechanisms, enabling the preferential translation of viral mRNA via an IRES-dependent mechanism that recruits ribosomes and ITAFs (41, 42). During FMDV infection, these ITAFs undergo important modifications to regulate virus life cycle, such as proteolytic cleavage by virus proteases, changes in phosphorylation levels, and redistribution from the nuclear to the cytoplasmic compartment. For example, DDX21 and DDX23 interact with FMDV IRES to diminish FMDV translation, but the inhibitory effect was antagonized by FMDV 3C^pro^ (10, 11). NCL, normally localized in the nucleus, is translocated to the cytoplasm during FMDV infection, where it promotes IRES-driven translation by facilitating the assembly of translation initiation complexes (12). HnRNP L negatively regulates FMDV replication by inhibiting viral RNA synthesis through interaction with FMDV IRES and 3D^pol^ (43). HnRNP K, as a novel ITAF, represses FMDV translation and replication, but hnRNP K-mediated inhibition was antagonized by FMDV 3C^pro^. Interestingly, the C-terminal cleavage product of hnRNP K becomes a positive regulator of FMDV replication, whereas the N-terminal product still retains partial inhibitory effects on IRES activity (44). Here, we found that the inhibitory effect of DDX5 was antagonized by FMDV 3ABCD protease precursor, rather than through 3C^pro^. Meanwhile, we co-transfected 3A, 3C^pro^, and 3D^pol^, but did not find any cleavage of DDX5. The result suggests that 3ABCD was expressed *in cis* with 3C^pro^ to cause the cleavage. Since the 3ABCD precursor can undergo autocatalytic processing to release mature viral proteins, including the 3C protease (Fig. 8E), we hypothesize that DDX5 cleavage likely occurs after its export from the nucleus and is mediated by the 3C protease released from the processed 3ABCD precursor. This hypothesis is strongly supported by our observation that the 3C protease alone neither triggers the relocalization of DDX5 nor cleaves it (Fig. 8D and F), indicating that the antagonism of FMDV against DDX5 is a multi-step process.

In conclusion, we have identified DDX5 as a novel restriction factor that negatively regulates FMDV translation and replication through binding to FMDV IRES and 3′UTR. Further research has shown that DDX5 hinders FMDV IRES-mediated translation by blocking the formation of the 80S ribosome. Additionally, we also found that DDX5 inhibits viral RNA synthesis via interacting with FMDV 3D^pol^. However, the antagonistic role of the FMDV 3ABCD protease precursor with autocatalytic processing on DDX5 remains to be further characterized. These insights into the mechanisms of DDX5-mediated restriction provide valuable information for understanding FMDV replication and could inform future antiviral strategies.

## Data Availability

Data sharing is not applicable to this article as no new data were created or analyzed in this study.

## SUPPLEMENTAL MATERIAL

Fig. S1 jvi.01731-25-s0001.tif Substitutions of GUAA and AAAA abrogated IRES-mediated translation activity.

Fig. S2 jvi.01731-25-s0002.tif DDX5 does not affect the translation of Cap-mediated rFMDV mCherry replicons.

Fig. S3 jvi.01731-25-s0003.tif The individual cleavage fragments of DDX5 do not have antiviral activity.

Supplemental legends jvi.01731-25-s0004.docx Descriptive legends for Fig. S1 to S3.

Table S1 jvi.01731-25-s0005.xlsx Identification of RNA-binding proteins that potentially interact with the FMDV 5'UTR.

Table S2 jvi.01731-25-s0006.xlsx Identification of RNA-binding proteins that potentially interact with the FMDV IRES.

ASM does not own the copyrights to Supplemental Material that may be linked to, or accessed through, an article. The authors have granted ASM a non-exclusive, world-wide license to publish the Supplemental Material files. Please contact the corresponding author directly for reuse.

## References

[1] Wu J, Sun C, Guan J, Abdullah SW, Wang X, Ren M, Qiao L, Sun S, Guo H. 2024. Nuclear ribonucleoprotein RALY downregulates foot-and-mouth disease virus replication but antagonized by viral 3C protease. Microbiol Spectr 12:e0365823. doi:10.1128/spectrum.03658-23 38323828 PMC10913732

[2] Li H, Liu P, Dong H, Dekker A, Harmsen MM, Guo H, Wang X, Sun S. 2024. Foot-and-mouth disease virus antigenic landscape and reduced immunogenicity elucidated in atomic detail. Nat Commun 15:8774. doi:10.1038/s41467-024-53027-5 39389971 PMC11467346

[3] Peng J, Yi J, Yang W, Ren J, Wen Y, Zheng H, Li D. 2020. Advances in foot-and-mouth disease virus proteins regulating host innate immunity. Front Microbiol 11:2046. doi:10.3389/fmicb.2020.02046 33162944 PMC7581685

[4] Aslam M, Alkheraije KA. 2023. The prevalence of foot-and-mouth disease in Asia. Front Vet Sci 10:1201578. doi:10.3389/fvets.2023.1201578 37456961 PMC10347409

[5] Li K, Wang C, Yang F, Cao W, Zhu Z, Zheng H. 2021. Virus-host interactions in foot-and-mouth disease virus infection. Front Immunol 12:571509. doi:10.3389/fimmu.2021.571509 33717061 PMC7952751

[6] Abdullah SW, Wu J, Wang X, Guo H, Sun S. 2023. Advances and breakthroughs in IRES-directed translation and replication of picornaviruses. mBio 14:e0035823. doi:10.1128/mbio.00358-23 36939331 PMC10127998

[7] Dong H, Liu P, Bai M, Wang K, Feng R, Zhu D, Sun Y, Mu S, Li H, Harmsen M, Sun S, Wang X, Guo H. 2022. Structural and molecular basis for foot-and-mouth disease virus neutralization by two potent protective antibodies. Protein Cell 13:446–453. doi:10.1007/s13238-021-00828-9 33599962 PMC9095805

[8] Lee KM, Chen CJ, Shih SR. 2017. Regulation mechanisms of viral IRES-driven translation. Trends Microbiol 25:546–561. doi:10.1016/j.tim.2017.01.010 28242053

[9] Walsh D, Mohr I. 2011. Viral subversion of the host protein synthesis machinery. Nat Rev Microbiol 9:860–875. doi:10.1038/nrmicro2655 22002165 PMC7097311

[10] Abdullah SW, Wu J, Zhang Y, Bai M, Guan J, Liu X, Sun S, Guo H. 2021. DDX21, a host restriction factor of FMDV IRES-dependent translation and replication. Viruses 13:1765. doi:10.3390/v13091765 34578346 PMC8473184

[11] Abdullah SW, Han S, Wu J, Zhang Y, Bai M, Jin Y, Zhi X, Guan J, Sun S, Guo H. 2020. The DDX23 negatively regulates translation and replication of foot-and-mouth disease virus and is degraded by 3C proteinase. Viruses 12:1348. doi:10.3390/v12121348 33255534 PMC7760909

[12] Han S, Wang X, Guan J, Wu J, Zhang Y, Li P, Liu Z, Abdullah SW, Zhang Z, Jin Y, Sun S, Guo H. 2021. Nucleolin promotes IRES-driven translation of foot-and-mouth disease virus by supporting the assembly of translation initiation complexes. J Virol 95:e0023821. doi:10.1128/JVI.00238-21 33853964 PMC8315980

[13] Han S, Sun S, Li P, Liu Q, Zhang Z, Dong H, Sun M, Wu W, Wang X, Guo H. 2020. Ribosomal protein L13 promotes IRES-driven translation of foot-and-mouth disease virus in a helicase DDX3-dependent manner. J Virol 94:e01679-19. doi:10.1128/JVI.01679-19 31619563 PMC6955262

[14] Song Y, Tzima E, Ochs K, Bassili G, Trusheim H, Linder M, Preissner KT, Niepmann M. 2005. Evidence for an RNA chaperone function of polypyrimidine tract-binding protein in picornavirus translation. RNA 11:1809–1824. doi:10.1261/rna.7430405 16314455 PMC1370870

[15] Belsham GJ, McInerney GM, Ross-Smith N. 2000. Foot-and-mouth disease virus 3C protease induces cleavage of translation initiation factors eIF4A and eIF4G within infected cells. J Virol 74:272–280. doi:10.1128/jvi.74.1.272-280.2000 10590115 PMC111537

[16] Devaney MA, Vakharia VN, Lloyd RE, Ehrenfeld E, Grubman MJ. 1988. Leader protein of foot-and-mouth disease virus is required for cleavage of the p220 component of the cap-binding protein complex. J Virol 62:4407–4409. doi:10.1128/JVI.62.11.4407-4409.1988 2845152 PMC253884

[17] Lawrence P, Schafer EA, Rieder E. 2012. The nuclear protein Sam68 is cleaved by the FMDV 3C protease redistributing Sam68 to the cytoplasm during FMDV infection of host cells. Virology (Auckl) 425:40–52. doi:10.1016/j.virol.2011.12.019 22280896

[18] Cheng W, Chen G, Jia H, He X, Jing Z. 2018. DDX5 RNA helicases: emerging roles in viral infection. Int J Mol Sci 19:1122. doi:10.3390/ijms19041122 29642538 PMC5979547

[19] Hu M, Zheng H, Wu J, Sun Y, Wang T, Chen S. 2022. DDX5: an expectable treater for viral infection- a literature review. Ann Transl Med 10:712. doi:10.21037/atm-22-2375 35845539 PMC9279824

[20] Li C, Ge L, Li P, Wang Y, Sun M, Huang L, Ishag H, Di D, Shen Z, Fan W, Mao X. 2013. The DEAD-box RNA helicase DDX5 acts as a positive regulator of Japanese encephalitis virus replication by binding to viral 3′ UTR. Antiviral Res 100:487–499. doi:10.1016/j.antiviral.2013.09.002 24035833 PMC7113685

[21] Xu J, Cai Y, Ma Z, Jiang B, Liu W, Cheng J, Guo N, Wang Z, Sealy JE, Song C, Wang X, Li Y. 2021. The RNA helicase DDX5 promotes viral infection via regulating N6-methyladenosine levels on the DHX58 and NFκB transcripts to dampen antiviral innate immunity. PLoS Pathog 17:e1009530. doi:10.1371/journal.ppat.1009530 33909701 PMC8081163

[22] López de Quinto S, Martínez-Salas E. 1997. Conserved structural motifs located in distal loops of aphthovirus internal ribosome entry site domain 3 are required for internal initiation of translation. J Virol 71:4171–4175. doi:10.1128/JVI.71.5.4171-4175.1997 9094703 PMC191578

[23] Martínez-Salas E, López de Quinto S, Ramos R, Fernández-Miragall O. 2002. IRES elements: features of the RNA structure contributing to their activity. Biochimie 84:755–763. doi:10.1016/s0300-9084(02)01408-6 12457563

[24] Han SC, Guo HC, Sun SQ. 2015. Three-dimensional structure of foot-and-mouth disease virus and its biological functions. Arch Virol 160:1–16. doi:10.1007/s00705-014-2278-x 25377637

[25] Rodríguez Pulido M, Sáiz M. 2017. Molecular mechanisms of foot-and-mouth disease virus targeting the host antiviral response. Front Cell Infect Microbiol 7:252. doi:10.3389/fcimb.2017.00252 28660175 PMC5468379

[26] Francisco-Velilla R, Embarc-Buh A, Abellan S, Martinez-Salas E. 2022. Picornavirus translation strategies. FEBS Open Bio 12:1125–1141. doi:10.1002/2211-5463.13400 PMC9157412 35313388

[27] Khan D, Fox PL. 2024. Host-like RNA elements regulate virus translation. Viruses 16:468. doi:10.3390/v16030468 38543832 PMC10976276

[28] Andreev DE, Niepmann M, Shatsky IN. 2022. Elusive trans-acting factors which operate with type I (Poliovirus-like) IRES elements. Int J Mol Sci 23:15497. doi:10.3390/ijms232415497 36555135 PMC9778869

[29] Johnson AG, Grosely R, Petrov AN, Puglisi JD. 2017. Dynamics of IRES-mediated translation. Philos Trans R Soc Lond B Biol Sci 372:20160177. doi:10.1098/rstb.2016.0177 28138065 PMC5311923

[30] Martínez-Salas E, Francisco-Velilla R, Fernandez-Chamorro J, Lozano G, Diaz-Toledano R. 2015. Picornavirus IRES elements: RNA structure and host protein interactions. Virus Res 206:62–73. doi:10.1016/j.virusres.2015.01.012 25617758

[31] Fuller-Pace FV. 2006. DExD/H box RNA helicases: multifunctional proteins with important roles in transcriptional regulation. Nucleic Acids Res 34:4206–4215. doi:10.1093/nar/gkl460 16935882 PMC1616952

[32] Ullah R, Li J, Fang P, Xiao S, Fang L. 2022. DEAD/H-box helicases:anti-viral and pro-viral roles during infections. Virus Res 309:198658. doi:10.1016/j.virusres.2021.198658 34929216

[33] Poynter SJ, Herrington-Krause S, DeWitte-Orr SJ. 2019. Two DExD/H-box helicases, DDX3 and DHX9, identified in rainbow trout are able to bind dsRNA. Fish Shellfish Immunol 93:1056–1066. doi:10.1016/j.fsi.2019.07.054 31340170

[34] Li F, Ling X, Chakraborty S, Fountzilas C, Wang J, Jamroze A, Liu X, Kalinski P, Tang DG. 2023. Role of the DEAD-box RNA helicase DDX5 (p68) in cancer DNA repair, immune suppression, cancer metabolic control, virus infection promotion, and human microbiome (microbiota) negative influence. J Exp Clin Cancer Res 42:213. doi:10.1186/s13046-023-02787-x 37596619 PMC10439624

[35] Fullam A, Schröder M. 2013. DExD/H-box RNA helicases as mediators of anti-viral innate immunity and essential host factors for viral replication. Biochim Biophys Acta 1829:854–865. doi:10.1016/j.bbagrm.2013.03.012 23567047 PMC7157912

[36] Taschuk F, Cherry S. 2020. DEAD-box helicases: sensors, regulators, and effectors for antiviral defense. Viruses 12:181. doi:10.3390/v12020181 32033386 PMC7077277

[37] Bonaventure B, Goujon C. 2022. DExH/D-box helicases at the frontline of intrinsic and innate immunity against viral infections. J Gen Virol 103. doi:10.1099/jgv.0.001766 36006669

[38] Zan J, Xu R, Tang X, Lu M, Xie S, Cai J, Huang Z, Zhang J. 2020. RNA helicase DDX5 suppresses IFN-I antiviral innate immune response by interacting with PP2A-Cβ to deactivate IRF3. Exp Cell Res 396:112332. doi:10.1016/j.yexcr.2020.112332 33065113

[39] Nie Z, Zhai F, Zhang H, Zheng H, Pei J. 2024. The multiple roles of viral 3D(pol) protein in picornavirus infections. Virulence 15:2333562. doi:10.1080/21505594.2024.2333562 38622757 PMC11020597

[40] Theerawatanasirikul S, Semkum P, Lueangaramkul V, Chankeeree P, Thangthamniyom N, Lekcharoensuk P. 2022. Non-nucleoside inhibitors decrease foot-and-mouth disease virus replication by blocking the viral 3Dpol Viruses 15:124. doi:10.3390/v15010124 36680163 PMC9866314

[41] Yang D, Sun C, Gao R, Wang H, Liu W, Yu K, Zhou G, Zhao B, Yu L. 2020. A temperature-dependent translation defect caused by internal ribosome entry site mutation attenuates foot-and-mouth disease virus: implications for rational vaccine design. J Virol 94:e00990-20. doi:10.1128/JVI.00990-20 32493820 PMC7394902

[42] Kieft JS. 2008. Viral IRES RNA structures and ribosome interactions. Trends Biochem Sci 33:274–283. doi:10.1016/j.tibs.2008.04.007 18468443 PMC2706518

[43] Sun C, Liu M, Chang J, Yang D, Zhao B, Wang H, Zhou G, Weng C, Yu L. 2020. Heterogeneous nuclear ribonucleoprotein L negatively regulates foot-and-mouth disease virus replication through inhibition of viral RNA synthesis by interacting with the internal ribosome entry site in the 5′ untranslated region. J Virol 94. doi:10.1128/JVI.00282-20 PMC7199413 32161169

[44] Liu W, Yang D, Sun C, Wang H, Zhao B, Zhou G, Yu L. 2020. hnRNP K is a novel internal ribosomal entry site-transacting factor that negatively regulates foot-and-mouth disease virus translation and replication and is antagonized by viral 3C protease. J Virol 94:e00803-20. doi:10.1128/JVI.00803-20 32581104 PMC7431795

