# Supplementary figures and images for: RNA helicase DDX5 regulates the translation and genomic replication of foot-and-mouth disease virus
Source: J Virol. 2026 Jan 30;100(3):e01731-25. doi: 10.1128/jvi.01731-25 (PMC13011465; doi:10.1128/jvi.01731-25)

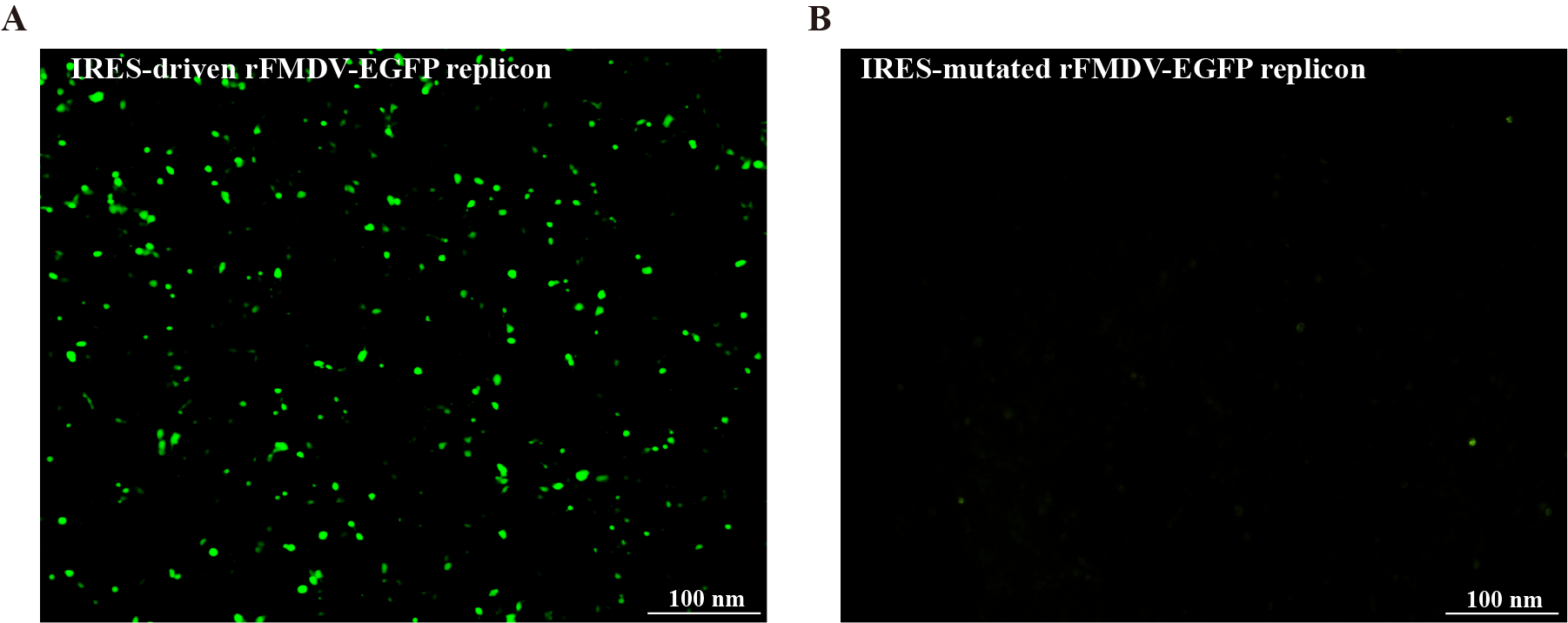

Supplement: Fig. S1 — Substitutions of GUAA and AAAA abrogated IRES-mediated translation activity. [file jvi.01731-25-s0001.tif]

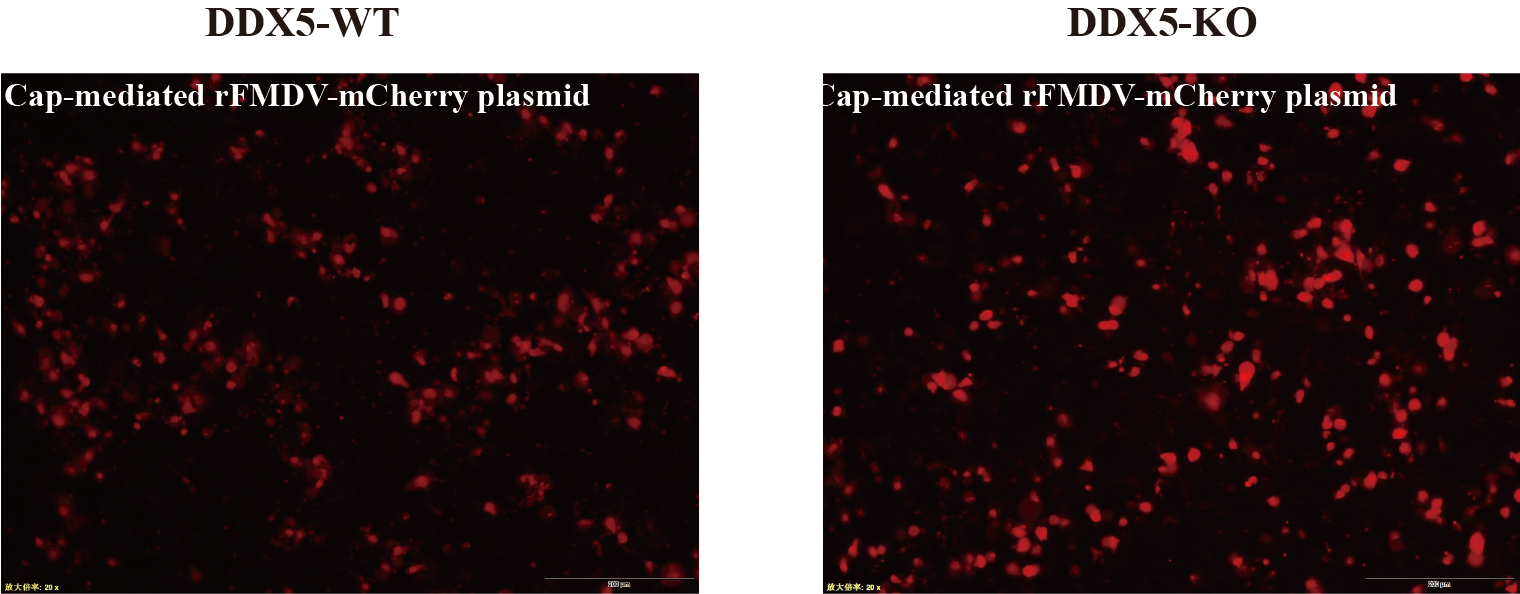

Supplement: Fig. S2 — DDX5 does not affect the translation of Cap-mediated rFMDV mCherry replicons. [file jvi.01731-25-s0002.tif]

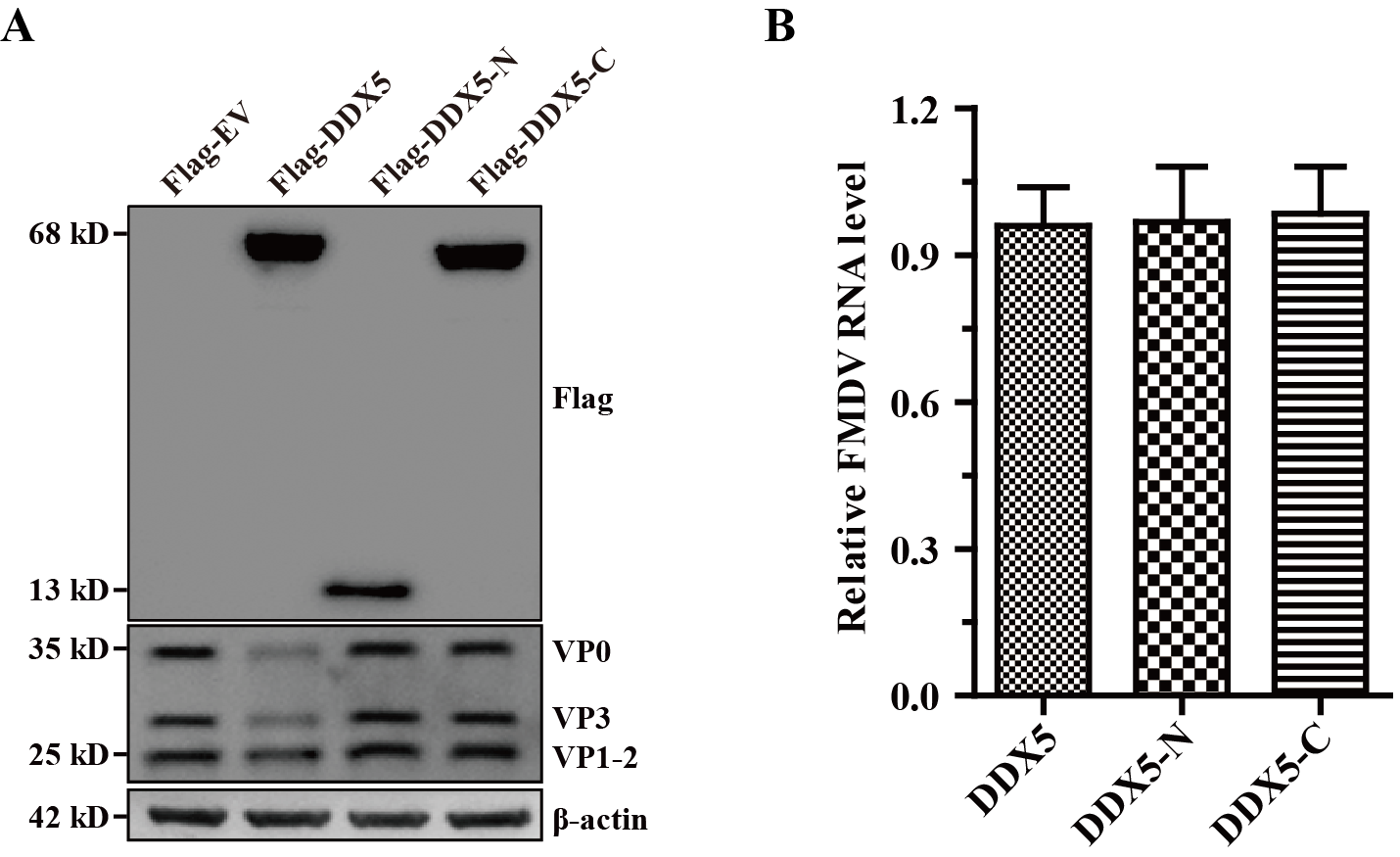

Supplement: Fig. S3 — The individual cleavage fragments of DDX5 do not have antiviral activity. [file jvi.01731-25-s0003.tif]
