# Supplementary material for: RNA helicase DDX5 regulates the translation and genomic replication of foot-and-mouth disease virus
Source: J Virol. 2026 Jan 30;100(3):e01731-25. doi: 10.1128/jvi.01731-25 (PMC13011465; doi:10.1128/jvi.01731-25)
Supplement: Supplemental legends — Descriptive legends for Fig. S1 to S3. [file jvi.01731-25-s0004.docx]

**Fig. S1** Substitutions of GUAA and AAAA abrogated IRES-mediated translation activity. (A) IRES-driven rFMDV replicon was transfected into PK-15 cells. EGFP was measured by fluorescence microscope (OLIMPUS, IX73, Japan). (B) IRES-mutated rFMDV replicon was transfected into PK-15 cells. The EGFP expression was measured by fluorescence microscope (OLIMPUS, IX73, Japan).

**Fig. S2** Cap-mediated rFMDV-mCherry plasmid was transfected into DDX5-WT and DDX5-KO cells, respectively. The mCherry expression was analyzed by fluorescence microscopy (Olympus IX73, Japan).

**Fig. S3** The individual cleavage fragments of DDX5 do not have antiviral activity. (A) Different DDX5 truncated fragments were transfected into PK-15 cells for 24 h, followed by FMDV infection. Cell lysates were harvested at 6 h and subjected to Western blotting analysis. (B) The relative FMDV RNA levels were analyzed by RT-qPCR.
